# GATA4-activated lncRNA MALAT1 promotes osteogenic differentiation through inhibiting NEDD4-mediated RUNX1 degradation

**DOI:** 10.1038/s41420-023-01422-0

**Published:** 2023-05-08

**Authors:** Xianzhe Huang, Shuo Jie, Wenzhao Li, Chan Liu

**Affiliations:** 1 Department of Orthopedics, The Second Xiangya Hospital, Central South University, Changsha, 410011 Hunan Province PR China; 2 International Medical Department, The Second Xiangya Hospital, Central South University, Changsha, 410011 Hunan Province PR China

**Keywords:** Cell growth, Bone cancer

## Abstract

Postmenopausal osteoporosis (PMOP) brings a lot of inconvenience to patients and serious economic burden to society. The osteogenic differentiation of bone marrow mesenchymal stem cells (BMSCs) plays vital role in the process of PMOP treatment. However, the functional mechanism remains unclear. In this study, GATA4, MALAT1 and KHSRP were downregulated in bone tissues of PMOP patients, while NEDD4 was overexpressed. Through functional experiments, GATA4 overexpression strikingly accelerated osteogenic differentiation of BMSCs and promoted bone formation in vitro and in vivo, while these effects were dramatically reversed after MALAT1 silence. Intermolecular interaction experiments confirmed that GATA4 activated the transcription of MALAT1, which could form a ‘RNA-protein’ complex with KHSRP to decay NEDD4 mRNA. NEDD4 promoted the degradation of Runx1 by ubiquitination. Moreover, NEDD4 silencing blocked the inhibitory effects of MALAT1 knockdown on BMSCs osteogenic differentiation. In sum up, GATA4-activated MALAT1 promoted BMSCs osteogenic differentiation via regulating KHSPR/NEDD4 axis-regulated RUNX1 degradation, ultimately improving PMOP.

## Introduction

The loss of structural elements and bone minerals caused by menopause affects more than 50% of women over 50 years of age, which suggests that postmenopausal osteoporosis (PMOP) has become a serious social public health problem [1]. Now, some therapeutic methods have been investigated to reduce the fracture risk of PMOP patients, however, a complete cure of PMOP remains a huge challenge. Bone marrow mesenchymal stem cells (BMSCs) are precursor cells of osteoblasts, indicating an important effect of BMSCs on bone formation. Increasing evidence demonstrated that the differentiation of BMSCs might be a vital step for treating PMOP [2]. Whereas, the specific molecular mechanism is still being explored. Long non-coding RNA (lncRNAs) are a class of endogenous single-stranded RNA, which can play as a critical regulator in PMOP [3], and in the process of BMSCs differentiation [4]. For instance, lncRNA MEG3 suppressed the osteogenic differentiation of BMSCs via directly binding miR-133a-3p, thus aggravating PMOP [5]. LncRNA metastasis-associated lung adenocarcinoma transcript 1 (MALAT1) was reported to be downregulated in osteoporosis, and BMSCs-derived exosomal MALAT1 promoted osteoblast activity in osteoporotic mice [6]. Furthermore, Li demonstrated that MALAT1 facilitated BMSC osteogenic differentiation and repressed macrophages osteoclastic differentiation during osteoporosis through miR-124-3p/IGF2BP1/Wnt/β-catenin axis [7]. Therefore, exploring the functional mechanism of MALAT1 may be a promising direction for the treatment of PMOP.

Ubiquitination is mediated by ubiquitin-activating enzymes (E1), ubiquitin-conjugating enzymes (E2), and ubiquitin-ligases (E3) and is a key posttranslational regulatory process [8]. NEDD4 family HECT-type E3 ubiquitin protein ligases showed functional effects on osteoblasts and their precursor cells through degrading a variety of critical regulators of bone genes, such as JUNB and Runt-related transcription factor 2 (Runx2) [9]. Runt domain transcription factors are mainly composed of Runx1, Runx2 and Runx3, which are important components of the cascade of bone differentiation-related signals [10]. Tang et al. suggested that Runx1 maintained adult bone homeostasis from bone loss though upregulating Bmp7/Alk3/Smad1/5/8/Runx2/ATF4 and WNT/β-Catenin signaling pathways [11]. Furthermore, a previous study demonstrated that NEDD4, the member of E3, downregulated Runx2 levels through facilitating the ubiquitination of Runx2 [12]. However, it is not clear whether NEDD4 can ubiquitin degrade Runx1.

LncRNAs function as archetypes of decoys, signals and scaffolds during the process of interaction with DNA, RNA or protein [13]. Previous reports indicated that lncRNAs regulated the stability of target mRNA and participated in the process of multiple disease by interacting with RNA-binding proteins (RBPs) [14, 15]. For instance, lncRNA RP11-138J23.1 was reported to participated in the progression of gastric cancer through enhancing the stability of VAV3 mRNA by targeting HuR protein [16]. K-homology splicing regulatory protein (KHSRP), as a RBP, was demonstrated to be vital in the process of mRNA metabolism. AU-rich elements (AREs) are specific base sequences that recognize and combine by KHSRP within the 3′-untranslated region (UTR) of certain mRNAs, thereby regulating their mRNA expression at a steady level [17]. StarBase (http://starbase.sysu.edu.cn/) [18] predicted a potential binding relationship among MALAT1, KHSRP and NEDD4. However, whether it exists in the differentiation of BMSCS has not been confirmed yet.

GATA binding protein 4 (GATA4) encodes a member of the GATA family of zinc-finger transcription factors which can recognize the GATA motif which is present in the promoters of many genes. Recently, GATA4 was reported to be essential for osteoblastic differentiation, bone remodeling and mineralization [19]. A previous study suggested that GATA4 play as a pioneer factor for estrogen receptor alpha (ERα) and a negative regulator of TGFβ signaling, and depletion of GATA4 resulted in perinatal mortality, decreased bone trabecular performance and abnormal bone development [20], indicating that GATA4 might be a potential target to develop therapies for PMOP. Moreover, JASPAR website (http://jaspar.genereg.net/) [21] indicated the high potential transcription recognition site of GATA4 on the promoter region of MATAL1. Thus, we speculated GATA4 might be regulated the transcription of MALAT1 to involve in bone differentiation.

In this study, we found the low levels of GATA4 and MALAT1 and high level of NEDD4 in bone tissues of PMOP patients and during the process of BMSCs differentiation. Overexpression of GATA4 promoted the osteoblastic differentiation through upregulating MALAT1. Mechanistically, MALAT1 reduced the stability of NEDD4 mRNA through binding to KHSRP, inhibiting the degradation of Runx1, thereby facilitating the osteoblastic differentiation. Our findings revealed a novel pathway GATA4/MALAT1/KHSRP/NEDD4 and provided a potential target for PMOP therapy.

## Results

### MALAT1 and GATA4 were downregulated in the bone tissues of PMOP patients and OVX mice

**Fig. 1:**
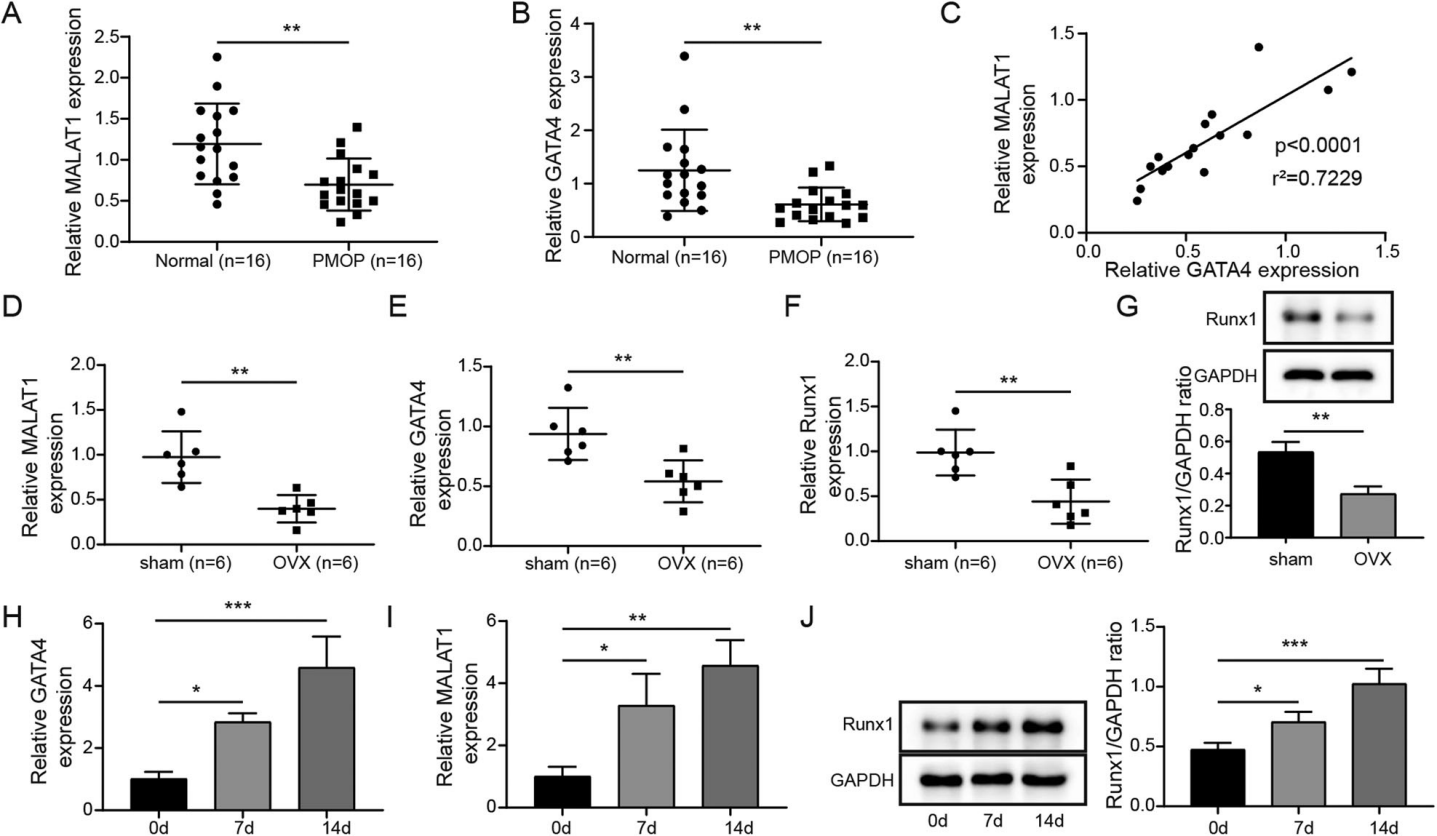
The downregulation of MALAT1 and GATA4 was observed in the bone tissues of PMOP patients and OVX mice. **A, B** The levels of MALAT1 and GATA4 in the bone tissues of PMOP patients were evaluated by qRT-PCR. *n* = 16. **C** The correlation of MALAT1 and GATA4 was analyzed by Pearson correlation coefficients. **D**, **E** The levels of MALAT1 and GATA4 in the bone tissues of mice treated with ovariectomy (OVX). **F**, **G** The mRNA and protein levels of Runx1 in the bone tissues of mice treated with OVX were measured by qRT-PCR and Western blotting. **H, I** Human BMSCs isolated from bone marrow specimens were cultured into osteogenic-inducing medium for 0, 7, 14 days, and then qRT-PCR analyzed MALAT1 and GATA4 levels in the process of osteogenic differentiation of BMSCs. **J** The protein expression of Runx1 in the process of osteogenic differentiation of BMSCs. Mean ± SD, *n* = 3, *p < 0.05, **p < 0.01, ***p < 0.001. Statistical analysis was carried out by a student t-test or a one-way ANOVA.

To examine the expressions of MALAT1 and GATA4, we collected the bone tissues from PMOP patients and OVX mouse model. Compared with those of normal bone tissues, MALAT1 and GATA4 levels were significantly decreased in bone tissues of PMOP patients (Fig. 1A, B). The data from Pearson correlation coefficients suggested that MALAT1 was positively correlated with GATA4 in clinical PMOP patients (Fig. 1C). Consistently, the low expression of MALAT1 and GATA4 were also observed in bone tissues obtained from OVX mice (Fig. 1D, E). The mRNA and protein levels of Runx1 in bone tissues of OVX mice was obviously reduced compared to that in sham group mice (Fig. 1F–G). In addition, human BMSCs isolated from bone marrow specimens were cultured into osteogenic-inducing medium for 0, 7, 14 days. Then, the expression of MALAT1, GATA4 and Runx1 were measured using qRT-PCR or western blot assays. The results indicated that MALAT1, GATA4 and Runx1 were all gradually elevated during osteogenic differentiation of BMSCs (Fig. 1H–J), suggesting the potential function of MALAT1 and GATA4 in PMOP.

### GATA4 positively regulated the osteogenic differentiation of BMSCs

**Fig. 2:**
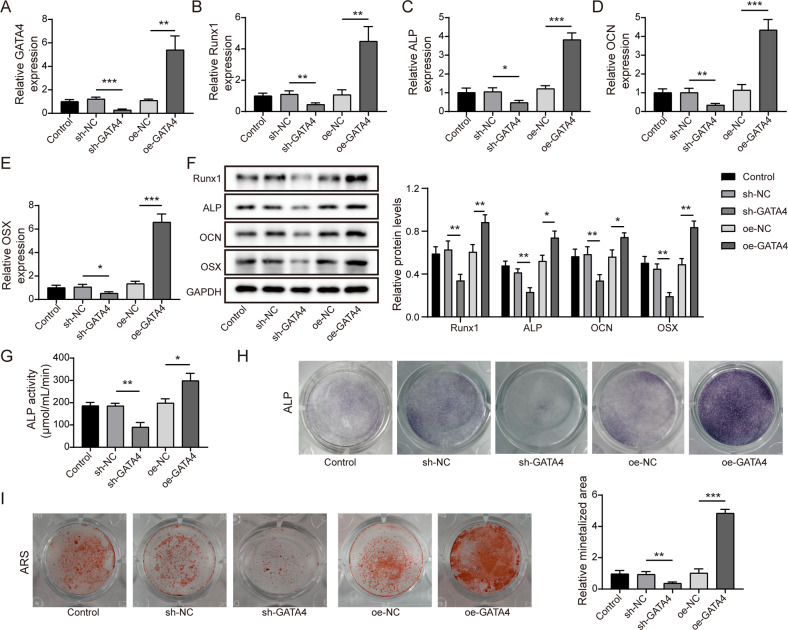
GATA4 promoted the differentiation of osteoblast in BMSCs. **A** The transfection efficiency of shGATA4 and oeGATA4 in BMSCs was determined by qRT-PCR. **B**–**F** The relative expression of Runx1, ALP, OCN and OSX was evaluated after silencing or overexpressing GATA4. **G** The ALP activity was measured by a test kit. **H** Representative images of ALP staining. **I** The osteogenic calcification was analyzed by Alizarin Red staining. Mean ± SD, *n* = 3, *p < 0.05, **p < 0.01, ***p < 0.001. Statistical analysis was carried out by one-way ANOVA.

We then began to analyze the biological effect of GATA4 bone differentiation in vitro. The shRNA and overexpressing plasmids of GATA4 were constructed and packaged by lentivirus, and followed by transfected into BMSCs. As shown in Fig. 2A, GATA4 level was significantly enhanced by oeGATA4 transfection and decreased by shGATA4 transfection. After osteogenic differentiation for 14 days, the mRNA expressions and protein levels of osteogenesis-related factors were evaluated by qRT-PCR and western blot analysis. The results showed that knockdown of GATA4 resulted in the downregulation of Runx1, ALP, OCN and OSX, while overexpression of GATA4 increased their levels (Fig. 2B–F). Moreover, ALP activity assay and ALP staining and Alizarin Red staining were performed to further identify the osteogenic differentiation of BMSCs. Compared to control group, ALP activity was reduced after silencing GATA4, while enhanced after overexpressing GATA4 (Fig. 2G–H). Similarly, knockdown of GATA4 alleviated the osteogenic calcification, while overexpression of GATA4 led to the opposite results (Fig. 2I). These data suggested that GATA4 was involved in the osteogenic differentiation process of BMSCs.

### GATA4 promoted the osteogenic differentiation through transcriptional activating MALAT1

**Fig. 3:**
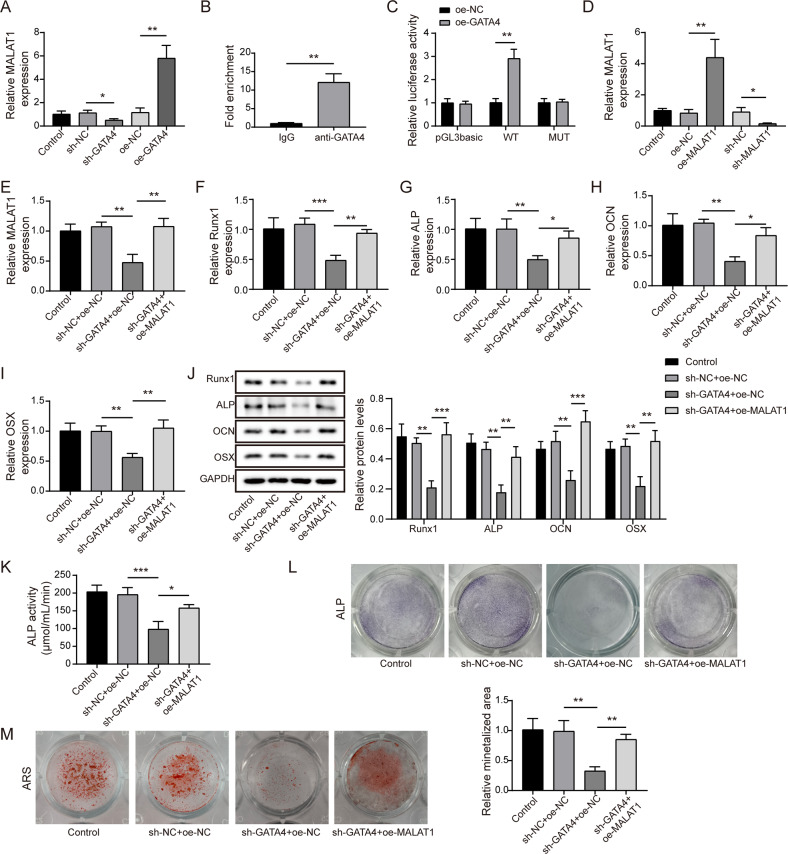
GATA4 promoted the osteogenic differentiation through transcriptional activating MALAT1. **A** The relative expression of MALAT1 was detected after silencing or overexpressing GATA4. **B**, **C** The interaction between GATA4 and MALAT1 promoter was evaluated by ChIP and Dual-luciferase reporter assays. **D** QRT-PCR analysis of MALAT1 expression after transfecting with oeMALAT1 or shMALAT1. **E** The effect of MALAT1 overexpression on the MALAT1 levels in cells transfected with shGATA4. **F**–**J** QRT-PCR and Western blotting were measured the levels of Runx1, ALP, OCN and OSX. **K** The ALP activity was measured by a test kit. **L** Representative images of ALP staining. **M** The osteogenic calcification was analyzed by Alizarin Red staining. Mean ± SD, *n* = 3, *p < 0.05, **p < 0.01, ***p < 0.001. Statistical analysis was carried out by one-way ANOVA.

Considering the positive correlation between GATA4 and MALAT1 in PMOP patients, it is reasonable to investigate the interaction and functional correlation of the two during osteogenic differentiation of BMSCs. We firstly observed that knockdown of GATA4 strikingly suppressed MALAT1 level, while overexpression of GATA4 greatly increased MALAT1 expression (Fig. 3A). Notably, we found a conserved transcription recognition site of GATA4 on MALAT1 promoter, which evidenced by JASPAR database. To confirm this prediction, ChIP assay was employed and indicated a significant enrichment of MALAT1 in complex immunoprecipitated by specific GATA4 antibody than IgG antibody (Fig. 3B). In addition, the luciferase activity in MALAT1-WT group was enhanced by overexpressing GATA4, while there was no obvious change in luciferase activity in MUT-MALAT1 group (Fig. 3C), suggesting GATA4 could directly bound to MALAT1 promoter. Next, a series rescue experiments were conducted to study the functional correlation between MALAT1 and GATA4. Evidencing by qRT-PCR, MALAT1 expression was remarkably inhibited after shMALAT1 transfection and markedly enhanced in the presence of oeMALAT1 (Fig. 3D). Moreover, we also observed that MALAT1 expression was inhibited by transfecting with shGATA4, while this inhibitory role was dramatically reversed by oeMALAT1 transfection (Fig. 3E). Interestingly, the downregulation of Runx1, ALP, OCN and OSX caused by silencing GATA4 was dramatically abolished through overexpressing MALAT1 (Fig. 3F–J). As expected, overexpression of MALAT1 greatly reversed the inhibiting effect of GATA4 knockdown on ALP activity and osteogenic calcification (Fig. 3K–M). Above all, it could be concluded that MALAT1 was a downstream transcriptional target of GATA4 in BMSCs osteogenic differentiation. Besides, to assess the relationship between osteoclast differentiation and GATA4 or MALAT1, THP-1 cells were transfected with shGATA4 or shMALAT1. As indicated in Fig. S1A–E, knockdown of GATA4 or MALAT1 increased the levels of osteoclastogenesis markers NFATc1, CtsK, C-src, and TRAP. Moreover, the data from TRAP staining suggested that GATA4 or MALAT1 knockdown resulted in significantly increased osteoclastogenesis (Fig. S1F). These findings demonstrated that GATA4 or MALAT1 might be negative players in osteoclast differentiation.

### MALAT1 decayed NEDD4 mRNA through binding KHSRP

**Fig. 4:**
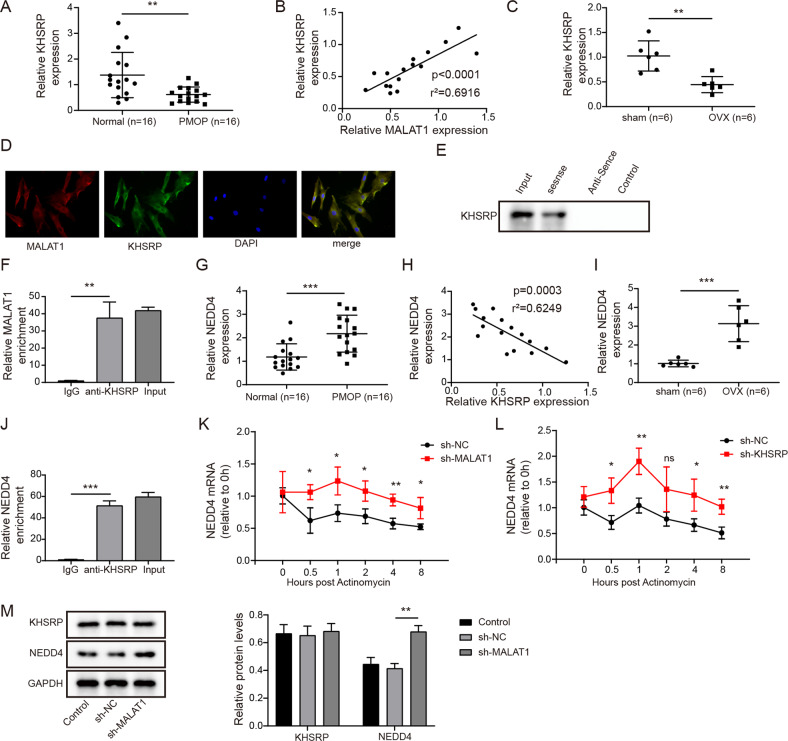
MALAT1 reduced the stability of NEDD4 mRNA through binding KHSRP. **A** Relative KHSRP level in bone tissues of PMOP patients. *n* = 16. **B** The correlation analysis of MALAT1 and KHSRP. **C** The expression of KHSRP in bone tissues of OVX mice. **D** FISH + immunofluorescence double staining indicated the co-localization of KHSRP (green) and MALAT1 (red). **E**, **F** RIP and RNA pull-down assays evaluated the interaction between MALAT1 and KHSRP. **G** The expression of NEDD4 in bone tissues of PMOP patients. *n* = 16. **H** The correlation analysis of KHSRP and NEDD4. **I** The expression of NEDD4 in bone tissues of OVX mice. **J** The analysis of targeted relationship between KHSPR and NEDD4. **K**, **L** Actinomycin D treatment to analyze the stability of NEDD4 mRNA in cells silenced MALAT1 or KHSRP. **M** The protein levels of KHSRP and NEDD4 in vitro were detected by Western blotting after knocking down MALAT1. Mean ± SD, *n* = 3, *p < 0.05, **p < 0.01, ***p < 0.001. Statistical analysis was carried out by one-way ANOVA.

This part was designed to excavate the underlying mechanism of MALAT1 during BMSCs osteogenic differentiation. Through starbase prediction, the potential molecular association among MALAT1, KHSRP and NEDD4 attracted our attention. Firstly, we measured the expression pattern of KHSRP in bone tissues of PMOP patients. As presented in Fig. 4A, KHSRP was greatly downregulated in bone tissues of PMOP patients compared to that in normal bone tissues. Moreover, a positive correlation was found in the expression of MALAT1 and KHSRP (Fig. 4B). Similarly, the downregulated KHSRP was also observed in bone tissues of OVX mice (Fig. 4C). In addition, the observation from the confocal microscope indicated significant co-localization between MALAT1 and KHSRP in the cytoplasm (Fig. 4D). The specific interaction between MALAT1 and KHSRP was also verified by RIP and RNA pull-down assays. RNA pull-down assay showed that KHSRP protein could be pulled down by biotin-labeled MALAT1 sense instead of MALAT1 antisense (Fig. 4E). Likewise, compared to IgG antibody, MALAT1 showed a significant abundance in RNA complex precipitated by KHSRP antibody (Fig. 4F). Furthermore, NEDD4 was suggested to be overexpressed in bone tissues of PMOP patients and OVX mice (Fig. 4G, I). Pearson correlation assay described a negative association between NEDD4 and MALAT1 in clinic expression (Fig. 4H). RIP experiment revealed that NEDD4 was presented a specific enrichment in sediments pulled down by KHSRP antibody, indicating an interaction relationship between them (Fig. 4J). Furthermore, the stability of NEDD4 mRNA was analyzed by Actinomycin D treatment after silencing MALAT1 or KHSRP. The results demonstrated that MALAT1 or KHSPR knockdown could enhance the mRNA stability of NEDD4 (Fig. 4K–L). Western blot assay demonstrated that knockdown of MALAT1 increased NEDD4 level, while the expression of KHSRP remained unchanged (Fig. 4M), suggesting that MALAT1 reduced the stability of NEDD4 mRNA through recruiting KHSRP.

### NEDD4 promoted the degradation of Runx1

**Fig. 5:**
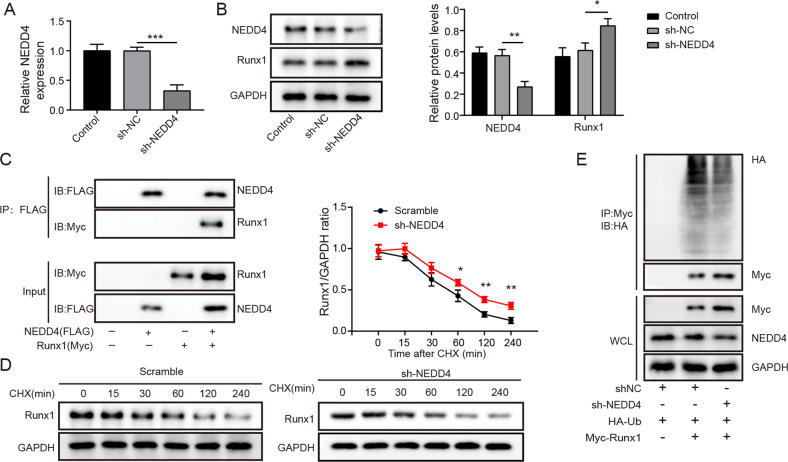
NEDD4 promoted the degradation of Runx1 by ubiquitination. **A** The knockdown efficiency of shNEDD4 was measured by qRT-PCR. **B** The protein levels of NEDD4 and Runx1 were detected by Western blotting after silencing NEDD4. **C** Co-IP used to analyze the interplay between NEDD4 and Runx1. **D** The turnover curve of Runx1 was measured in cells treated with cycloheximide (CHX). **E** Co-IP analysis of Myc-tagged Runx1 with Flag-tagged NEDD4. Mean ± SD, *n* = 3, *p < 0.05, **p < 0.01, ***p < 0.001. Statistical analysis was carried out by one-way ANOVA.

NEDD4, a E3 ubiquitin protein ligase, was thought to be the major regulators of Runx2 poly-ubiquitination [22]. Whereas, whether NEDD4 play a similar regulatory role in Runx1 remain elusive. Firstly, the shRNA targeting NEDD4 was transfected into BMSCs, and greatly reduced the expression of NEDD4 (Fig. 5A). Western blot assay illustrated that knockdown of NEDD4 significantly reduced NEDD4 protein level but enhanced Runx1 protein level (Fig. 5B). Moreover, the data from Co-IP assay indicated that Runx1 was well detected in Flag-labeled NEDD4 significantly immunoprecitated Runx1 (Fig. 5C). Cells were treated with CHX after transfection with shNEDD4, as shown in Fig. 5D, CHX treatment promoted the degradation of Runx1, while NEDD4 knockdown significantly prevented this effect. Moreover, in vitro ubiquitination experiment showed that silencing of NEDD4 greatly reduced the ubiquitin modification level and enhanced Runx1 protein levels (Fig. 5E). Taken together, NEDD4 facilitated the degradation of Runx1 by ubiquitination.

### Knocking down of MALAT1 inhibited the osteogenic differentiation of BMSCs through enhancing NEDD4

**Fig. 6:**
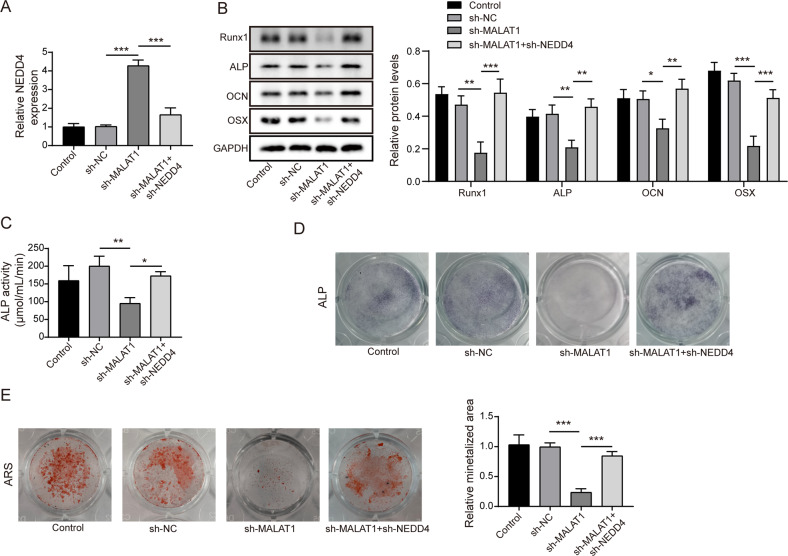
MALAT1 promoted the osteogenic differentiation through inhibiting NEDD4 by targeted upregulating Runx1. Cells were divided into four groups: Control, shNC, shMALAT1 and shMALAT1 + shNEDD4. **A** The mRNA expression of NEDD4 in different groups. **B** The expression of Runx1, ALP, OCN and OSX. **C** The analysis of ALP activity in different groups. **D** Representative images of ALP staining. **E** The analysis of osteogenic calcification. Mean ± SD, *n* = 3, *p < 0.05, **p < 0.01, ***p < 0.001. Statistical analysis was carried out by one-way ANOVA.

After confirming the interaction of MALAT1/NEDD4 and NEDD4/Runx1, we began to explore the biological function of this novel axis in BMSCs. Firstly, shMALAT1 was transfected into BMSCs alone or co-transfected with shNEDD4. Then, we observed that the expression levels of NEDD4 were increased after silencing MALAT1, while co-transfection with shNEDD4 could reverse these changes (Fig. 6A). Moreover, silencing of MALAT1 significantly reduced the levels of osteogenic differentiation markers such as Runx1, ALP, OCN and OSX, but these roles were dramatically abolished by co-downregulation of NEDD4 (Fig. 6B). Additionally, the reduction of ALP activity caused by MALAT1 knockdown in BMSCs was further enhanced through silencing NEDD4 (Fig. 6C, D). Similarly, knockdown of NEDD4 obviously exacerbated the osteogenic calcification, which remarkably reversed the inhibitory role mediated by MALAT1 silencing (Fig. 6E). Thus, these data suggested that MALAT1 silence reduced BMSCs osteogenic differentiation by regulating NEDD4/Runx1 axis.

### GATA4 facilitated the bone formation in OVX mice by upregulating MALAT1

**Fig. 7:**
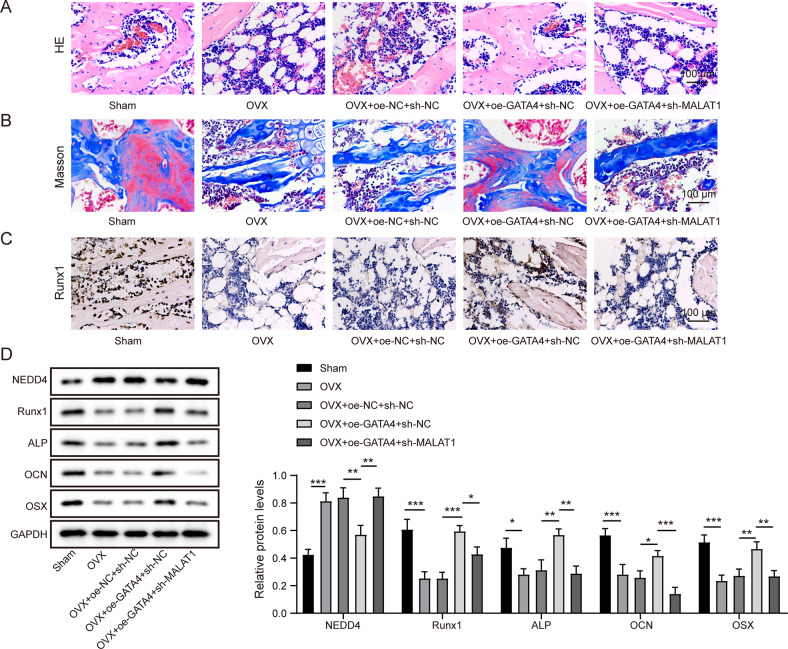
GATA4 facilitated the bone formation in OVX mice by upregulating MALAT1. Mice were divided into five groups: Sham, OVX, OVX + oeNC+shNC, OVX + oeGATA4+shNC and OVX + oeGATA4 + shMALAT1 (*n* = 6). **A, B** Histological assessment of ectopic bone formation through HE and Masson staining. **C** Representative images of IHC staining to evaluate the expression of Runx1. **D** The expression of NEDD4, Runx1, ALP, OCN and OSX was measured by Western blotting. Mean ± SD, *p < 0.05, **p < 0.01, ***p < 0.001. Statistical analysis was carried out by one-way ANOVA.

To further confirm the functional correlation between GATA4 and MALAT1 in vivo, OVX mice was injected with the following lentivirus vectors: oeNC + shNC, oeGATA4 + shNC and oeGATA4 + shMALAT1, through the tail vein. H&E and Masson staining were adopted to assess the bone formation. As shown in Fig. 7A, B, compared to sham group, the number of trabecular bones and collagen tissue were reduced in OVX mice, and GATA4 overexpression significantly increased the number of trabecular bones and collagen tissue in OVX mice, however, this effect was strikingly reversed after MALAT1 silence. Immunohistochemical results demonstrated that GATA4 overexpression facilitated the expression of Runx1 in bone tissues of OVX mice, while downregulation of MALAT1 restrained this trend (Fig. 7C). Furthermore, the osteogenic-related markers in bone tissues of OVX mouse model were identified using western blot assay. Compared to sham group, high expression of NEDD4 and the low expression of Runx1, ALP, OCN and OSX were observed in OVX mice (Fig. 7D). On these basis, overexpression of GATA4 remarkably decreased NEDD4 level and enhanced Runx1, ALP, OCN and OSX levels, while MALAT1 silencing reversed these changes (Fig. 7D). In addition, bone tissue was subjected to TRAP staining. The results suggested that overexpression of GATA4 reduced TRAP-positive cells in OVX mice, while MALAT1 knockdown blocked this effect (Fig. S1G). Collectively, these findings indicated that GATA4 contributed to the bone formation in vivo by increasing MALAT1 levels.

## Discussion

PMOP patients have low bone mineral density and a high risk of fracture [23]. Osteogenic differentiation was reported to be contributed to bone remodeling [24]. The quantity and function of BMSCs have a direct impact on the number and role of osteoblasts. In the study of PMOP, BMSCs viability was reported to be suppressed, and the ability of ALP and calcified nodule induced was reduced in vitro [25]. Estrogen deficiency was a leading cause for PMOP, and it treatment could facilitate the proliferation and osteogenic differentiation of hBMSCs via enhancing Notch pathway, thus effectively protect PMOP [26]. An another study also highlighted that osteogenic differentiation of BMSCs could help prevent osteoporosis in OVX mice [27]. Therefore, exploring the mechanism of promoting osteogenic differentiation of BMSCs is a promising method to treat PMOP. In this study, we found that GATA4 transcription activated MALAT1 to promote the osteogenic differentiation of BMSCs in vitro and the bone formation of OVX mice, which might be achieved through KHSRP/NEDD4/Runx1 axis.

GATA4 is a cardiomyocyte-specific zinc transcription factor that regulates differentiation, growth, and survival [28]. Recently, GATA4 was reported to be a target of estrogen receptor alpha [29], which was essential for osteoblastic differentiation, bone remodeling and mineralization [19]. A previous study showed that rapamycin facilitated the osteogenic differentiation of human blood-derived stem cells through enhancing transcription factors associated with bone formation such as GATA4, Otx2, Snail and Sox17 [30]. Zhou et al. also uncovered that GATA4 silencing could decrease the ALP activity and formation of calcified nodule, as well as bone formation in during tooth movement [19]. The similarly role of GATA4 was also confirmed by Khalid et al’ study, loss of GATA4 by cre-recombinase could decrease trabecular number, and increase trabecular spacing, reduce osteoblast cell number but increase osteoclast number, which mechanism seems to be associated with the activation of RANKL [31]. In the present work, we observed low expression of GATA4 in PMOP patients and OVX mice. Whereas, the expression of GATA4 increased during osteogenic differentiation of BMSCs. Overexpression of GATA4 could promote the osteogenic differentiation of BMSCs and bone formation and collagen tissue accumulation via enhancing ALP activity, calcified nodules formation and osteogenic markers in vitro and in vivo. Thus, it could be concluded GATA4 might be a protective factor in PMOP.

LncRNAs, more than 200 nucleotides in length, have key roles in many aspects of life, including dosage compensation, epigenetic control, and cell differentiation regulation [32]. And the related pathways of lncRNAs were suggested to regulate the process of osteogenic differentiation of BMSCs. MALAT1 was found to be downregulated in patients with PMOP, and MALAT1 levels were negatively associated with severity of PMOP [33]. Song et al. suggested that MALAT1 expression was suppressed in hindlimb unloading mice and simulated microgravity treated cells, and overexpression of MALAT1 alleviated osteoporosis through regulating miR-217/AKT3 axis [34]. Furthermore, the role of MALAT1 in promoting osteogenic differentiation has been reported in a variety of cells, such as human periodontal ligament stem cells [35], renal interstitial fibroblasts [36], and BMSCs [7]. Gao et al. demonstrated that MALAT1 knockdown reduced the expression of OCN, ALP, and OSX during the osteogenic differentiation of human BMSCs [37]. Consistently, our results also found that MALAT1 was significantly downregulated in bone tissues of PMOP patients and gradually overexpressed in BMSCs osteogenic differentiation. Besides, we found the positive correlation between MALAT1 and GATA4 in PMOP patients and investigated the transcriptional regulatory relationship of them. Through functional experiments, it was confirmed in vitro and in vivo that MALAT1 knockdown blocked the protective effects of GATA4 overexpression on the osteogenic differentiation of BMSCs and the bone formation of OVX mice. To our best knowledge, this study was the first to confirm the biological functional correlation between MALAT1 and GATA4. Moreover, we observed that GATA4 or MALAT1 inhibited osteoclast differentiation.

The subcellular localization of lncRNAs was closely related to their functions. LncRNAs influence transcriptional programs in the nucleus through chromatin interactions and remodeling, as well as establishing spatial structure of the nuclear compartment through scaffolding [38]. In the cytoplasm, lncRNAs were suggested to sequester miRNAs or proteins to modulate their expression, affect protein posttranslational modifications or mRNA stability [39, 40]. MALAT1 was involved in multiple diseases by interacting with miRNA or proteins [41, 42]. Here, we observed the co-localization of MALAT1 and KHSRP in the cytoplasm. As an RBP, KHSRP was suggested to interact with lncRNAs in different tumors, and these interactions were involved in the regulation of downstream genes stability [43, 44]. Notably, a previous report showed that KHSRP was dysregulated in MSCs and monocytes of PMOP patients, and presented the promising value for diagnosing and treating PMOP [45]. Here, our results further confirmed the low expression of KHSRP in PMOP patients and OVX mice. RIP and RNA pull-down assays verified a molecular interaction among MALAT1 KHSRP and NEDD4. Moreover, knockdown of MALAT1 or KHSRP enhanced the stability of NEDD4 mRNA. From these data, we verified the molecular regulation mechanism of MALAT1/KHSRP/NEDD4 axis in PMOP for the first time, and further elucidated the underlying mechanism of MALAT1 in regulating BMSCs osteogenic differentiation and PMOP therapy.

Ubiquitination was first discovered as a trigger for protein breakdown by the 26S proteasome, but it now has many additional proteasome-independent activities, such as signaling and selective autophagy [46, 47]. Ubiquitination therefore affects the majority of cellular processes and is crucial for a plethora of physiological processes, including cell survival and differentiation and innate and adaptive immunity. An increasing amount of data suggests that NEDD4 family HECT-type E3 ubiquitin protein ligases degrade a variety of critical regulators of bone anabolism in osteoblasts and their progenitors [22]. For example, lncRNA SNHG1 overexpression facilitated the ubiquitination of p-p38 via regulating NEDD4 [48]. This publication also suggested that knockdown of NEDD4 could increase ALP activity and OSX levels, which was consistent with our findings. NEDD4, as a member of NEDD4 family and a ubiquitinating enzyme, ubiquitin degraded Runx2, thereby affecting the bone formation in PMOP [12]. Thus, the similar mechanism was investigated between NEDD4 and Runx1. As our showed, NEDD4 could directly bind to Runx1 to inhibiting its protein ubiquitination, thus elevating protein stability. Runx1 maintained adult bone homeostasis and bone differentiation though upregulating Bmp7/Alk3/Smad1/5/8/Runx2/ATF4 and WNT/β-Catenin signaling pathways, suggesting that targeting Runx1 might be a novel therapeutics for osteoporosis [11]. Besides, our findings demonstrated that NEDD4 knockdown reversed the suppression effect of MALAT1 knockdown on the osteogenic differentiation of BMSCs. We therefore concluded that for the first time MALAT1 upregulated Runx1 through inhibiting NEDD4, thereby promoting the osteogenic differentiation of BMSCs.

In conclusion, we have proved a novel mechanism by which MALAT1 overexpressing by GATA4 could increase Runx1 through binding to KHSRP/NEDD4 signaling axis, thus promoting the osteogenic differentiation of BMSCs, ultimately contributing to bone formation in PMOP. These findings provided novel insights into potential mechanisms for the development of PMOP and might explore an effective approach to improve PMOP.

## Materials and methods

### Clinical samples

Trabecular bone tissues were collected from 16 patients with PMOP (female, age 55–75) and 16 patients suffered with external traumatic fracture (female, age 52–73, used for negative controls) at the Second Xiangya Hospital, Central South University from November 2020 to October 2021. Bone marrow samples were obtained from these patients. All samples were frozen at a −80 °C. The study protocol was approved by the Ethics Committee of the Second Xiangya Hospital, Central South University. All the patients have signed the informed consent before participating in this study. Inclusion criteria: PMOP patients: the bone density T-score −2.5; normal patients: hospitalized for trauma and the bone density T-score 1. Exclusion criteria: (1) Osteoporosis caused by other diseases such as diabetes and hyperthyroidism; (2) Used drugs affecting bone metabolism within six months; (3) Ovariectomized patients.

### Isolation of BMSCs and induction of osteogenic differentiation

Referencing the previous publication [49], human BMSCs were isolated from the bone marrow specimens of patients suffered with external traumatic fracture, and then purified by Percoll density gradient centrifugation method (GE Healthcare Life Sciences). BMSCs with CD90, CD44 and CD29 positive, as well as CD34, CD45 and CD14 negative, were selected for experiments. BMSCs were detected to be mycoplasma free. BMSCs from passages 3 to 5 were used in this study and cultured in DMEM/F12 (Invitrogen, Carlsbad, CA, USA) supplemented with 10% FBS (Invitrogen) and 1% penicillin-streptomycin solution. The medium was changed every 3 days. Then, BMSCs (5 × 10^5^) seeded in six-well plates were growth in a medium containing 100 nM dexamethasone (Sigma-Aldrich, CA, USA), 10 mM β-glycerophosphate (Sigma-Aldrich) and 0.2 mM ascorbic acid (Sigma-Aldrich) to induce osteogenic differentiation. The following tests were performed on 0d, 7d and 14d.

### THP-1 cell culture and induction of osteoclast differentiation

Human monocytic cells THP-1 purchased from American Type Culture Collection (Rockville, USA) were cultured in Roswell Park Memorial Institute (RPMI) 1640 medium with 10% FBS, supplemented with 100 U/ml penicillin, 100 μg/ml streptomycin and 0.25 μg/ml fungizone. The cell lines were authenticated by STR DNA profiling analysis and tested for mycoplasma contamination. For osteoclast differentiation, cells were first incubated with 100 ng/mL M-CSF (macrophage-colony stimulating factor) for 2 days to recruit macrophages. Afterward, cells were cultured in RANKL (50 ng/mL) for 12 days.

### Cell transfection

For the lentivirus-mediated knockdown assays, specific short hairpin RNAs (shRNAs) targeted GATA4, MALAT1, KHSRP or NEDD4 were inserted into pGLVH1 vector (GenePharm, China) individually to establish recombinant pGLVH1 knockdown vectors (shGATA4, shMALAT1, shNEDD4). Moreover, the packaged GATA4 or MALAT1-overexpressing lentivirus vector were obtained from Cyagen Biosciences (CA, USA). All of the above recombinant lentivirus vectors were co-transfected with packing plasmids (pGag/Pol, GenePharm) into 293 T cells. After 72 h of transfection, the culture medium supernatant was harvested and the virus concentration was adjusted to 30–40 MOI. BMSCs were plated into 24-well plates at a concentration of 5 × 10^5^ cells/well. Infection was performed when the BMSCs reached to 40% confluence in the presence of puromycin (5 μg/ml, Invitrogen). After 48 h of infection with lentivirus, the knockdown or overexpression efficiency of indicated lentivirus was verified by qRT-PCR.

### Establishment of ovariectomized (OVX) mice model

C57BL/6 mice (8 weeks old, female) were obtained from m SLAC Laboratory Animal Company, Ltd (Shanghai, China). All mice were housed in condition with humidity and temperature stability, and with a light–dark cycle time of 12 h. Mice were randomly divided into two groups: Sham group (*n* = 6, removed the fat around the ovary through operation), and OVX mice group (*n* = 32, removed the ovaries). After anesthesia, back skin and muscle were incised along the spine of the mouse. Ovaries and surrounding adipose tissue were removed in OVX group, and only removed periovarian adipose tissues in sham group. Then, the abdominal incisions were carefully sutured in both groups. Afterwards, OVX mice then were randomly divided into four groups (*n* = 8/per group): OVX, OVX + oeNC + shNC, OVX + oeGATA4 + shNC, and OVX + oeGATA4 + shMALAT1. According to experimental group, OVX mice were injected with the corresponding lentivirus-packed plasmid or shRNAs via the tail vein. After 4 weeks, mice were euthanized, and bone tissues were harvested for further study. All animal experiments were approved by Institutional Animal Care and Use Committee of the Second Xiangya Hospital, Central South University. The investigator was blinded to the group allocation during the experiment.

### Measurement of alkaline phosphatase (ALP)

After BMSCs osteogenic differentiation for 14 days, the ALP concentration of treated cells or tissues was evaluated by an ALP assay kit (Jiancheng Bioengineering Institute, Nanjing, China) according to the protocols. For ALP staining, cells were harvested and washed with PBS for 3 times. Then, cells were fixed with 4% paraformaldehyde, followed by stained using an ALP staining kit supplied by Beyotime (Shanghai, China). The stained image was observed and captured under a microscope (Nikon Corporation, Tokyo, Japan).

### Alizarin Red S staining (ARS)

ARS staining was conducted to evaluate the calcium nodule formation after osteogenic differentiation for 14 days. Briefly, BMSCs cells were collected and fixing with 4% paraformaldehyde, cells were stained with 1% ARS staining solution (Sigma-Aldrich, St Louis, MO, USA) for 30 min. Afterwards, cells were washed by PBS and analyzed under a microscope (Nikon Corporation).

### Fluorescence in situ hybridization (FISH) and immunofluorescence staining co-staining

The MALAT1 FISH probes were designed and supplied by RiboBio (Guangzhou, China). According to manufacturer’s instructions of FISH kit (RiboBio), cells were harvested and fixed with 4% paraformaldehyde. Then, cells were treated with 0.5% Triton X-100, and followed by incubated with the specific MALAT1 probe (1: 50) for overnight. Next, the cells were incubated with anti-KHSRP (1:200, ab150393, Abcam) for overnight, followed by the incubation with Alexa fluor 488-conjugated secondary antibody (ab150073, Abcam, Cambridge, UK) for 2 h. Cells were further stained with 4′, 6-diamidino-2-phenylindole (DAPI) to label cell nucleus. Finally, cells were observed and photographed under a confocal microscope (Olympus, Japan).

### Chromatin immunoprecipitation (ChIP)-PCR

The commercial ChIP kit were purchased from Millipore (Bedford, MA, USA) to validate the molecular relationship between GATA4 and the promoter region of MALAT1. Anti-IgG (ab182931, Abcam) was used as negative control, anti-GATA4 (ab256782, Abcam) was used to capture target DNA. Briefly, cells were crosslinked with 1% formaldehyde at 37 °C for 10 min, and glycine was added to terminate the crosslinking. After washing by PBS, the cells were resuspended with SDS lysis buffer, and then ultrasonically broken. The ultrasonic extract was incubated overnight with anti-IgG and anti-GATA4 at 4 °C. Afterwards, the precipitated protein-DNA complex was collected and crosslinked at 65 °C. After treatment with RNaseA and protease K, DNA samples were collected for PCR analysis.

### Dual-luciferase reporter assay

The binding sequence between MALAT1 promoter and GATA4 was predicted by JASPAR website (http://jaspar.genereg.net/). A MutaBest kit (Takara, Shiga, Japan) was used to mutate GATA4 recognition site in MALAT1 promoter region. The wild type (WT) or mutant type (MUT) sequence of MALAT1 promoter (WT/MUT-MALAT1) were inserted into a pGL3-Basic control vector (Promega, WI, USA). To validate the binding relationship between MALAT1 and GATA4, cells were co-transfected with either recombinant luciferase reporter constructs with oeGATA4 or oeNC by using Lipofectamine 3000. At 48 h after transfection, the luciferase activities were determined with Dual-Luciferase Reporter Assay System (Promega).

### Quantitative real-time PCR (qRT-PCR) analysis

TRIzol reagent (Invitrogen) was employed to extract the total RNAs of treated cells. For extracting RNAs from bone tissues of mice, frozen bones wrapped by foil were refrozen in liquid nitrogen and then ground into a powder using a hammer, followed by extracting RNAs using TRIzol reagent (Invitrogen). RNAs were then reversely transcribed into cDNA using PrimeScript™ RT Kit (Takara, Japan) and qRT-PCR processes were conducted using SYBR Green kit (Roche, Switzerland) on an ABI 7500 Real-Time System (Applied Biosystems). GAPDH was used as internal control. The primers were provided in Table 1.

**Table 1:** The primers used for qRT-PCR.

Gene name	Primer name	Primer sequence (5′ to 3′)
MALAT1	Human_ MALAT1_ F	GGACTACAGAGCCCCGAATT
	Human_ MALAT1_R	CCCTGCGTCATGGATTTCAAG
MALAT1	Mouse_ MALAT1_ F	GTATGTAGGCCTTTGCGGGT
	Mouse_ MALAT1_R	GGTTGTGCTGGCTCTACCAT
GATA4	Human_GATA4_F	CAGTCTACGTGCCCACACC
	Human_GATA4_R	TCCCGCCTGGCTCCAT
GATA4	Mouse_GATA4_F	CTGTGCCAACTGCCAGACTA
	Mouse_GATA4_R	TTTGAATCCCCTCCTTCCGC
NEDD4	Human_ NEDD4_F	TCAGGACAACCTAACAGATGCT
	Human_ NEDD4_R	TTCTGCAAGATGAGTTGGAACAT
NEDD4	Mouse_ NEDD4_F	GGAGTCTTTGGATATTGTAAGAGC
	Mouse_ NEDD4_R	GAGCGTGCGCCTCACAAGTATGA
RUNX2	Human_RUNX2_F	CGGAATGCCTCTGCTGTTAT
	Human_ RUNX2_R	TTCCCGAGGTCCATCTACTG
RUNX2	Mouse_ RUNX2_F	AGATGGGACTGTGGTTACCG
	Mouse_ RUNX2_R	GGACCGTCCACTGTCACTTT
ALP	Human_ALP_F	AACCCCAGACCCTGAGTACC
	Human_ ALP_R	CATGAGATGGGTCACAGACG
OCN	Human_OCN_F	GGCAGCGAGGTAGTGAAGAG
	Human_OCN_R	CTAGACCGGGCCGTAGAAG
OSX	Human_OSX_F	GGCACAAAGAAGCCGTACTC
	Human_OSX_R	CACTGGGCAGACAGTCAGAA
KHSRP	Human _KHSRP_F	AGATCAACCGGAGAGCAAGA
	Human _KHSRP_R	TCCTGTCAAGGACACACTGC
NFATc1	Human _NFATc1_F	GTCAGAGCGAGACTCAGAGG
	Human _NFATc1_R	CGGAGGAAAGTCATCGAGGG
Ctsk	Human _Ctsk_F	TTCCCGCAGTAATGACACCC
	Human _Ctsk_R	ACCCACAGAGCTAAAAGCCC
C-src	Human _C-src_F	CCCTTTCCCCTCTAGCCTCA
	Human _C-src_R	CAGTAGGCACCTTTCGTGGT
TRAP	Human _TRAP_F	CTTTCTACCGCCTGCACTTC
	Human _TRAP_R	GTTTCTTGAGCCAGGACAGC
GAPDH	Human_GAPDH_F	CTGACTTCAACAGCGACACC
	Human_GAPDH_R	GTGGTCCAGGGGTCTTACTC
GAPDH	Mouse_ GAPDH _F	AGCCCAAGATGCCCTTCAGT
	Mouse_ GAPDH _R	CCGTGTTCCTACCCCCAATG

### Western blot analysis

Proteins from cells and tissues were prepared using RIPA lysis buffer (Thermo Fisher Scientific, Waltham, MA, USA), BCA kit (Beyotime Biotechnology, Shanghai, China) was adopted to determine the protein concentration. Equal protein samples were separated by loading into 10% SDS-PAGE and followed by transferred onto PVDF membranes. Then, the membranes were blocked with 5% non-fat milk for 1 h. After washing, the membranes were incubated with primary antibodies against Runx1 (ab240639, Abcam), ALP (ab229126, Abcam), Osteocalcin (OCN, ab133612, Abcam), Osterix (OSX, ab209484, Abcam), KHSRP (ab150393, Abcam), NEDD4 (ab240753, Abcam), NFATc1 (ab25916, Abcam), Ctsk (ab187647, Abcam), C-src (ab185617, Abcam), and TRAP (ab52750, Abcam) and GAPDH (ab9485, Abcam) at 4 °C for 24 h. After washing, the membranes were further incubated with corresponding horseradish peroxidase-conjugated secondary antibodies for another 2 h. Finally, the band was detected with enhanced chemiluminescence reagent (Beyotime), and the band signals were analyzed through Image J software.

### mRNA stability assay

The total RNA of BMSCs were extracted using TRIzol reagent (Invitrogen). Then, the total RNA was exposed to actinomycin D (10 mg/ml; Sigma-Aldrich) for 0, 4, 8, 12 and 24 h. The relative abundance of NEDD4 was measured by qRT-PCR.

### RNA pull-down assay

RNA pull-down was performed to analyze the interplay between MALAT1 and KHSRP. Biotin-labeled MALAT1 was transcribed by using a Bitotin RNA labeling and T7 RNA polymerase (Roche, Basel, Switzerland). Bitotin labeled MALAT1 was incubated with streptavidin-conjugated magnetic beads (Invitrogen) overnight at 4 °C to generate beads-RNA complex. Then, the complex was added into cell lysates containing protease/phosphate Inhibitor for 4 h at 4 °C. The beads complex was harvested and wash thoroughly. Finally, the pulled down proteins were extracted and examined using western blot.

### RNA immunoprecipitation (RIP)

RIP assay was conducted to analyze the interaction between KHSRP and NEDD4 or MALAT1 using the Magna RIP kit (Millipore) following the manufacturer’s instructions. Cells were harvested and lysed in the complete RIP lysis buffer. Then, lysate was incubated with magnetic beads conjugated with anti-KHSRP (ab264265, Abcam) or IgG (ab205718, Abcam). Co-precipitated RNAs were extracted and detected using qRT-PCR assay.

### Co-immunoprecipitation (Co-IP) assay

Plasmids of pCMV-Myc-Runx1 and p3*Flag-NEDD4 provided by GenePharma (Shanghai, China) were co-transfected into BMSCs using Lipofectamine 3000 reagent (Invitrogen). Post-transfection for 48 h, the relationship between NEDD4 and Runx1 was detected by co-immunoprecipitation kit (Thermo Fisher Scientific) according to kit’s instruction. Briefly, BMSCs were collected and lysed at ice-cold IP buffer at 4 °C for 10 min. After centrifugation, the cell extracts were harvested and incubated with the primary antibodies against Myc (Sigma-Aldrich) or Flag (Sigma-Aldrich) for 0.5 h at 4 °C and followed by added 20uL Protein A/G Agarose (Millipore) for incubation 3 h at 4 °C. Precipitated complex was collected and washed with lysis buffer for 4 times. Then, the immunoprecipitated protein was then added SDS-PAGE sample buffer and boiled 5 min at 100 °C. Afterwards, the protein signals were determined utilizing western blot assay as described above.

### In vitro ubiquitination analysis

To test the ubiquitination of target substrate by NEDD4, the Ubiqutin Conjugation intiation Kit supplied by Boston Biochem (Boston, MA, USA) was used for in vitro ubiquitination assay. Te mixed reaction reagents containing 100 nM E1 Enyme, 1 µM E2 UbcH5b and, 5 µM ubiquitin-Flag to 10 µl of the ligase/substrate fraction for around half hour. The reaction products were incubated with corresponding Protein A/G magnetic beads which were pre-incubated with specific ubiquitination antibody. The non-substrate conjugated ubiquitin was removed by washing with cell lysis buffer for at least three times. Then, resuspended in 6.25 µl SDS-PAGE sample buffer for electrophoresis.

### Detection of protein stability

The protein synthesis inhibitor cycloheximide (CHX, Sigma-Aldrich) was transfected with cells to examine the protein stability of Runx1. Briefly, cells were incubated with CHX (100 μg/ml) for 0, 15, 30, 60, 120 240 min. Then, the total protein was extracted and determined by western blot.

### Hematoxylin and eosin (H&E) staining

The tissue samples were collected and fixed in 4% paraformaldehyde for around 30 min. Then, the samples were washed thoroughly with PBS and embedded in paraffin, and a microtome was used to cut the samples into 4 µm sections. After dewaxing and dehydrating, slices were stained with hematoxylin and eosin. Three high power fields (200 ×) were randomly selected per section to check under a light microscope (Olympus, Tokyo, Japan).

### Masson staining

Sample sections were prepared as described in HE staining. Cell nucleuses were stained by Wiegert’s iron haematoxylin solution (Sigma-Aldrich) for 10 min. Following thoroughly rinsing with PBS for 3 times, the tissue sections were dyed by Masson-Ponceau-acid fuchsin solution (0.7%, Sigma-Aldrich) for around 10 min. Sections were then rinsed in 2% glacial acetic acid and differentiated with phosphomolybdic acid for 4 min, followed by the staining with 2% aniline blue dye reagent (Sigma-Aldrich). The sections were imaged using a light microscope (Olympus, Tokyo, Japan) after dehydrating by ethanol series.

### Tartrate-resistant acid phosphatase (TRAP) staining

Treated THP-1 cells or bone sample sections were fixed and then stained with TRAP staining solution (Sigma-Aldrich) at 37 °C for 1 h. The stained cells were imaged with an inverted microscope (×100). Cells were considered to be osteoclasts if they were TRAP-positive multi nucleated cells with more than 3 nuclei

### Immunohistochemistry (IHC)

Sections were dewaxed and rehydrated, antigen retrieval was then performed by high-pressure heat. Afterwards, sections were incubated with 0.3% hydrogen peroxide for blockage, and then subjected for incubation with anti-Runx1 antibody (ab92336, abcam) overnight at 4 °C and followed by incubated with a secondary antibody (ab150116, abcam) temperature. The sections were then stained with diaminobenzidine (DAB) (Carpinteria, USA) for 5 min, washed in water, and counterstained in hematoxylin. Finally, the result was photographed under a microscope.

### Statistical analysis

All experiments were performed for at least three times, and data was presented as mean ± standard deviation. All data were in a normal distribution, and variance was similar between the groups that are being statistically compared. Data was organized and analyzed in Graphpad Prism (Version 7.0, USA). And student’s *t* test or one-way ANOVA followed by Tukey’s post-hoc test was adopted to compare the difference between two or multiple groups, respectively. *P* value less than 0.05 was considered significant.

## Supplementary information

Supplementary figure legend

Supplementary figure 1

Original Data File

## Data Availability

All data generated or analyzed during this study are included in this published article.
