# Supplementary material for: GATA4-activated lncRNA MALAT1 promotes osteogenic differentiation through inhibiting NEDD4-mediated RUNX1 degradation
Source: Cell Death Discov. 2023 May 8;9:150. doi: 10.1038/s41420-023-01422-0 (PMC10167365; doi:10.1038/s41420-023-01422-0)
Supplement: Supplementary file 1 — Supplementary figure legend [file 41420_2023_1422_MOESM1_ESM.docx]

**Supplementary Figure 1. GATA4 and MALAT1 were vital regulators of osteoclastogenesis *in vitro* and *in vivo*.** **(A-E)** qRT-PCR and Western blotting analysis of NFATc1, CtsK, C-src, and TRAP levels in THP-1 cells transfected with shGATA4 or shMALAT1. **(F)** The differentiation of THP-1 cells into osteoclasts was assessed by TRAP staining. **(G)** TRAP staining of bone tissues of OVX mice injected with oeGATA4 or oeGATA4+shMALAT1. Mean ± SD, n=3, *p < 0.05, **p < 0.01, ***p < 0.001. Statistical analysis was carried out by one-way ANOVA.
