# Supplementary figures and images for: GATA4-activated lncRNA MALAT1 promotes osteogenic differentiation through inhibiting NEDD4-mediated RUNX1 degradation
Source: Cell Death Discov. 2023 May 8;9:150. doi: 10.1038/s41420-023-01422-0 (PMC10167365; doi:10.1038/s41420-023-01422-0)

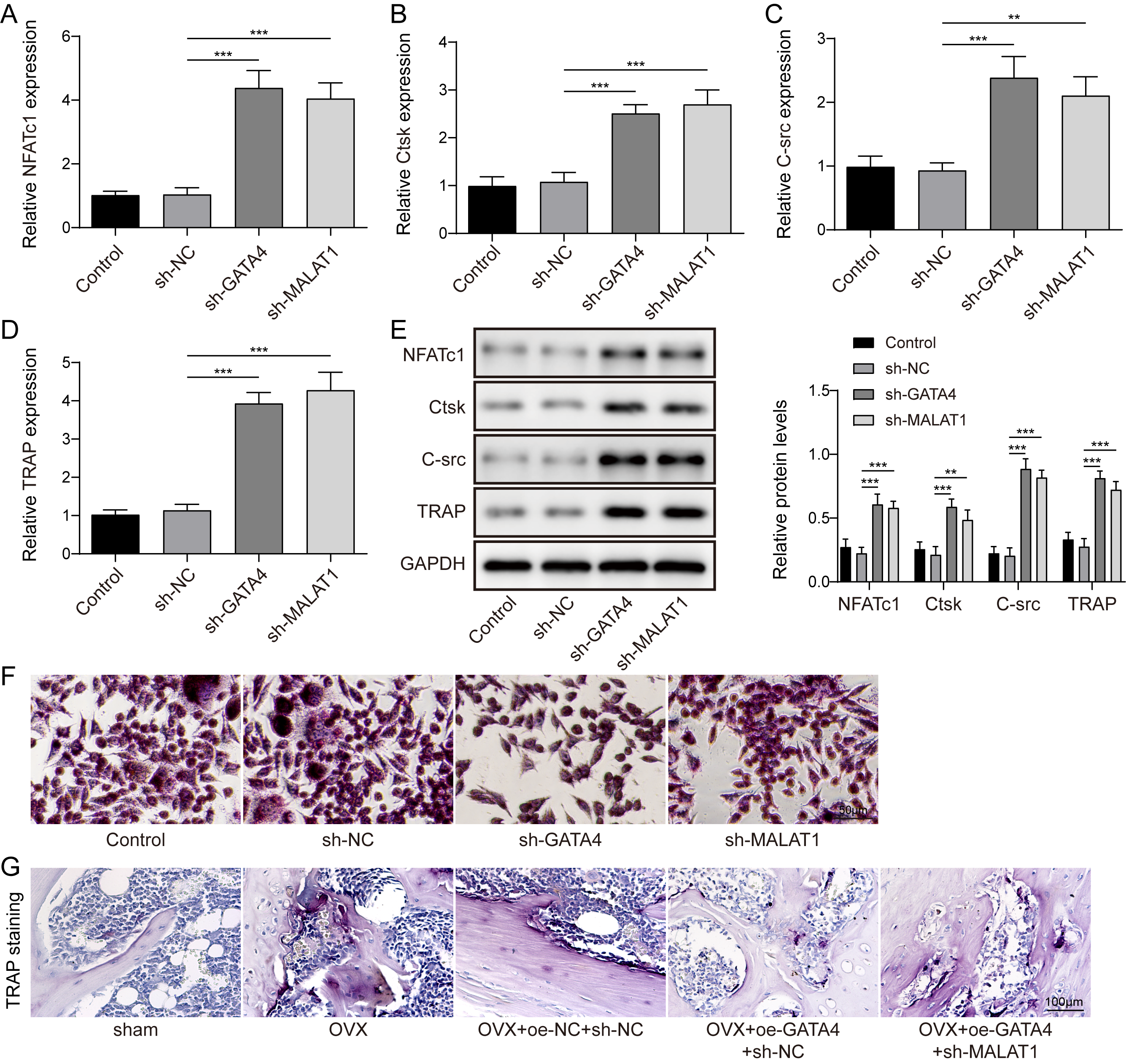

Supplement: Supplementary file 2 — Supplementary figure 1 [file 41420_2023_1422_MOESM2_ESM.tif]

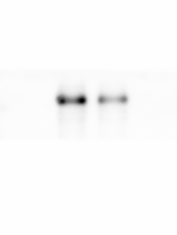

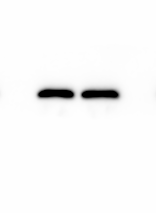

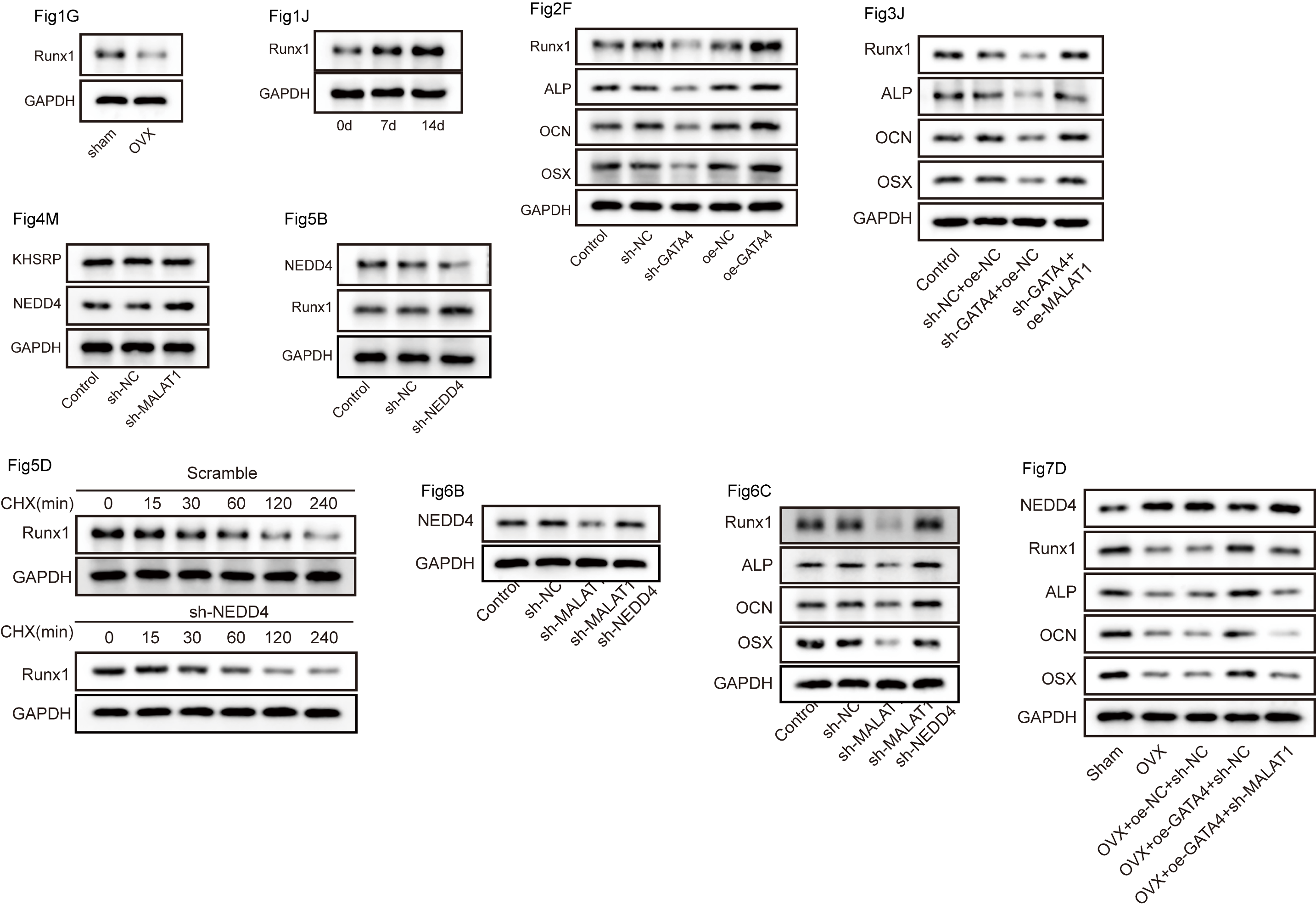


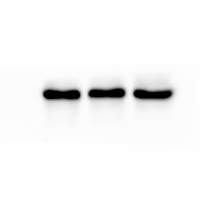

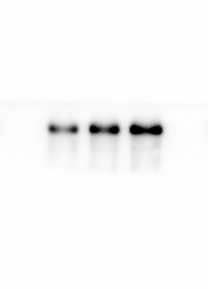

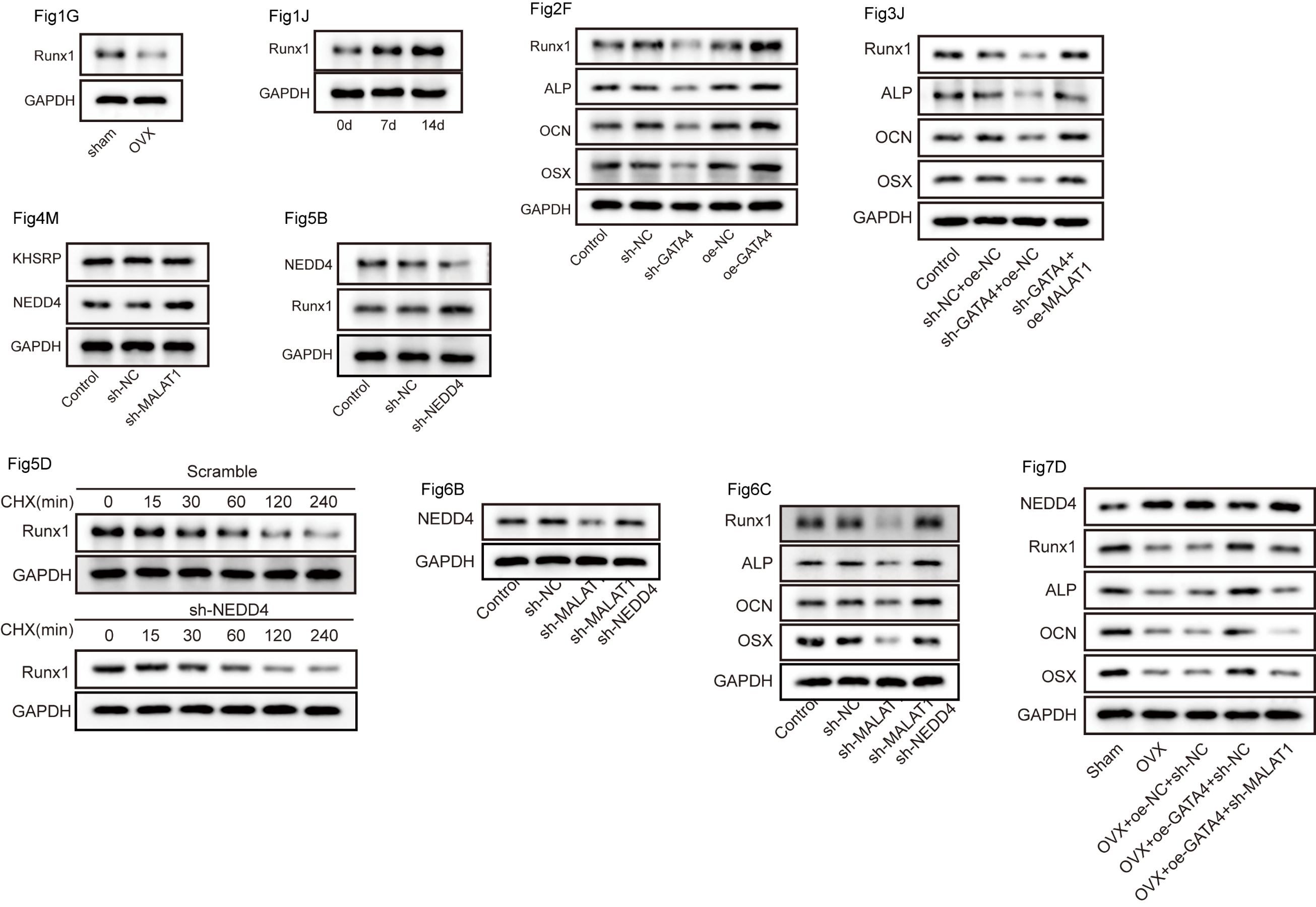


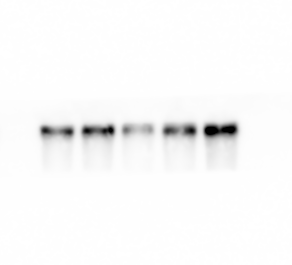


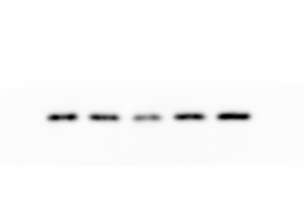


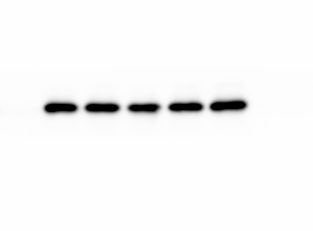

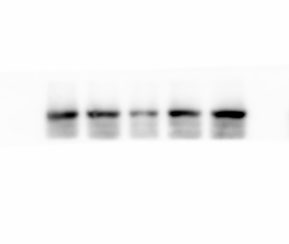

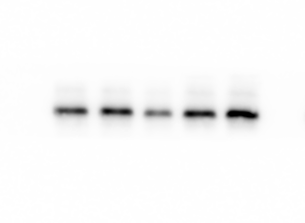

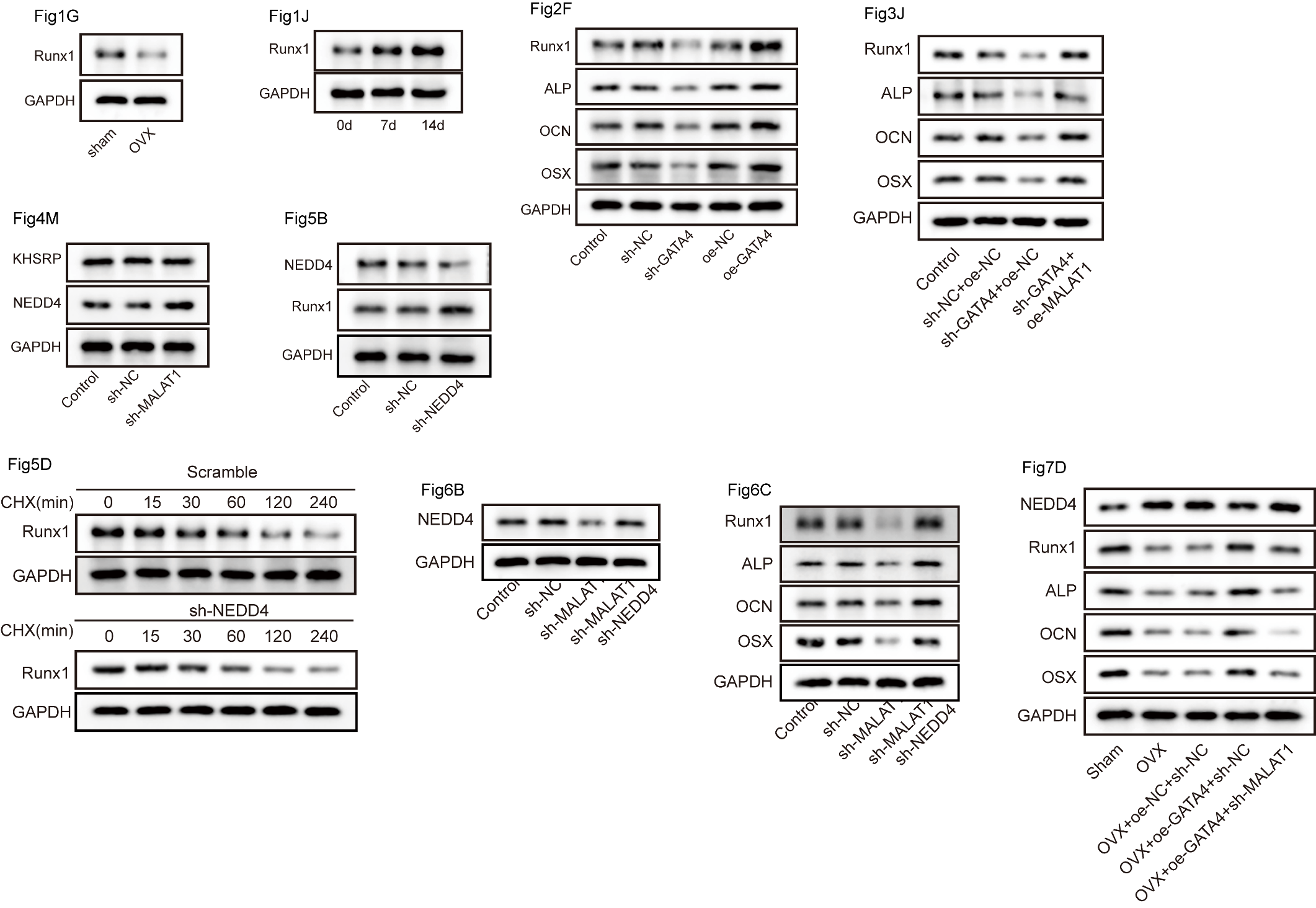


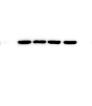

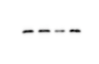

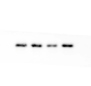

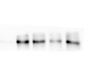

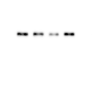

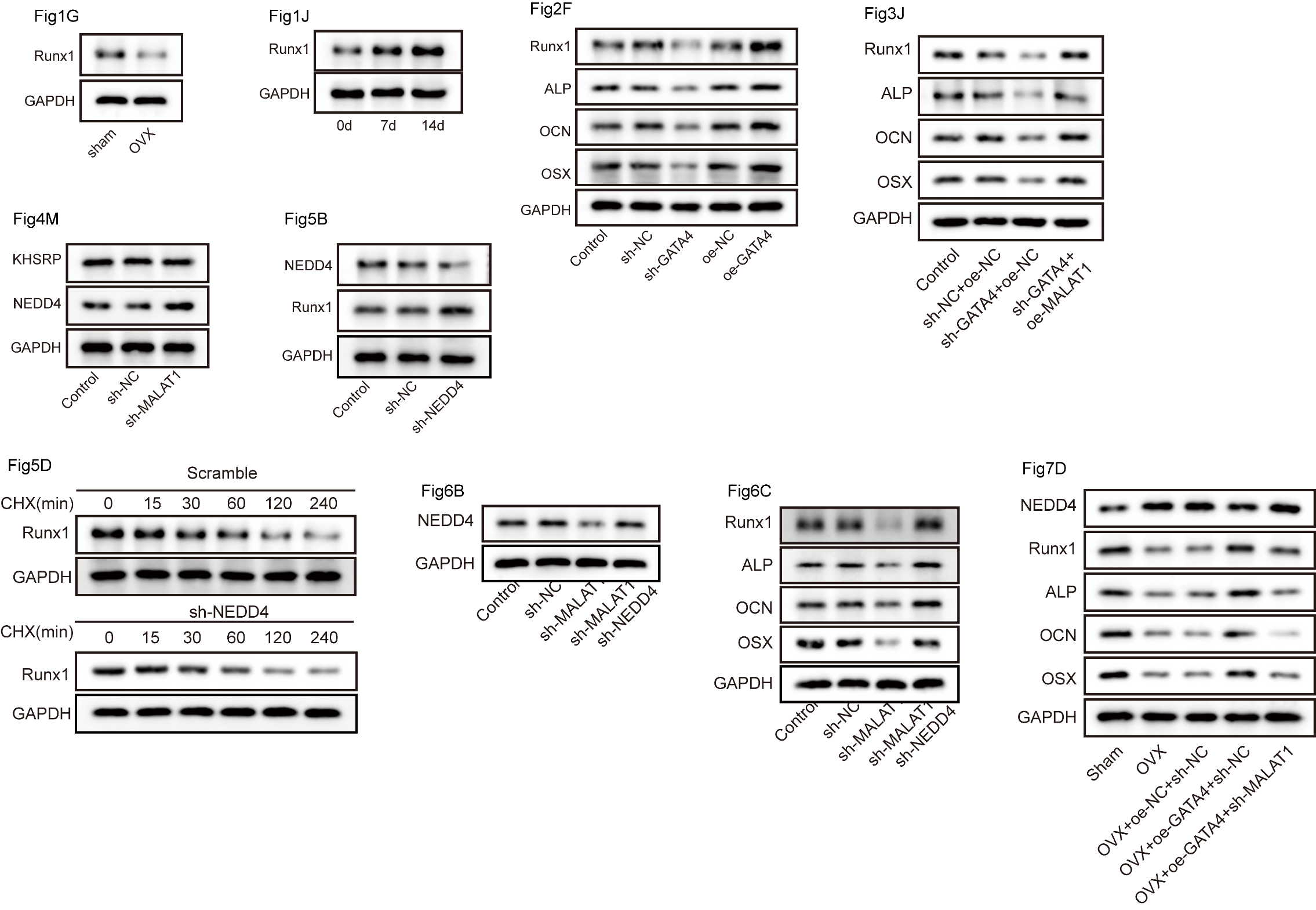


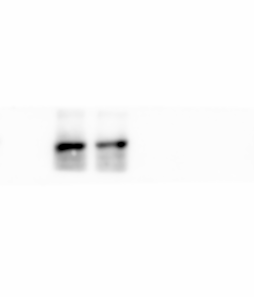

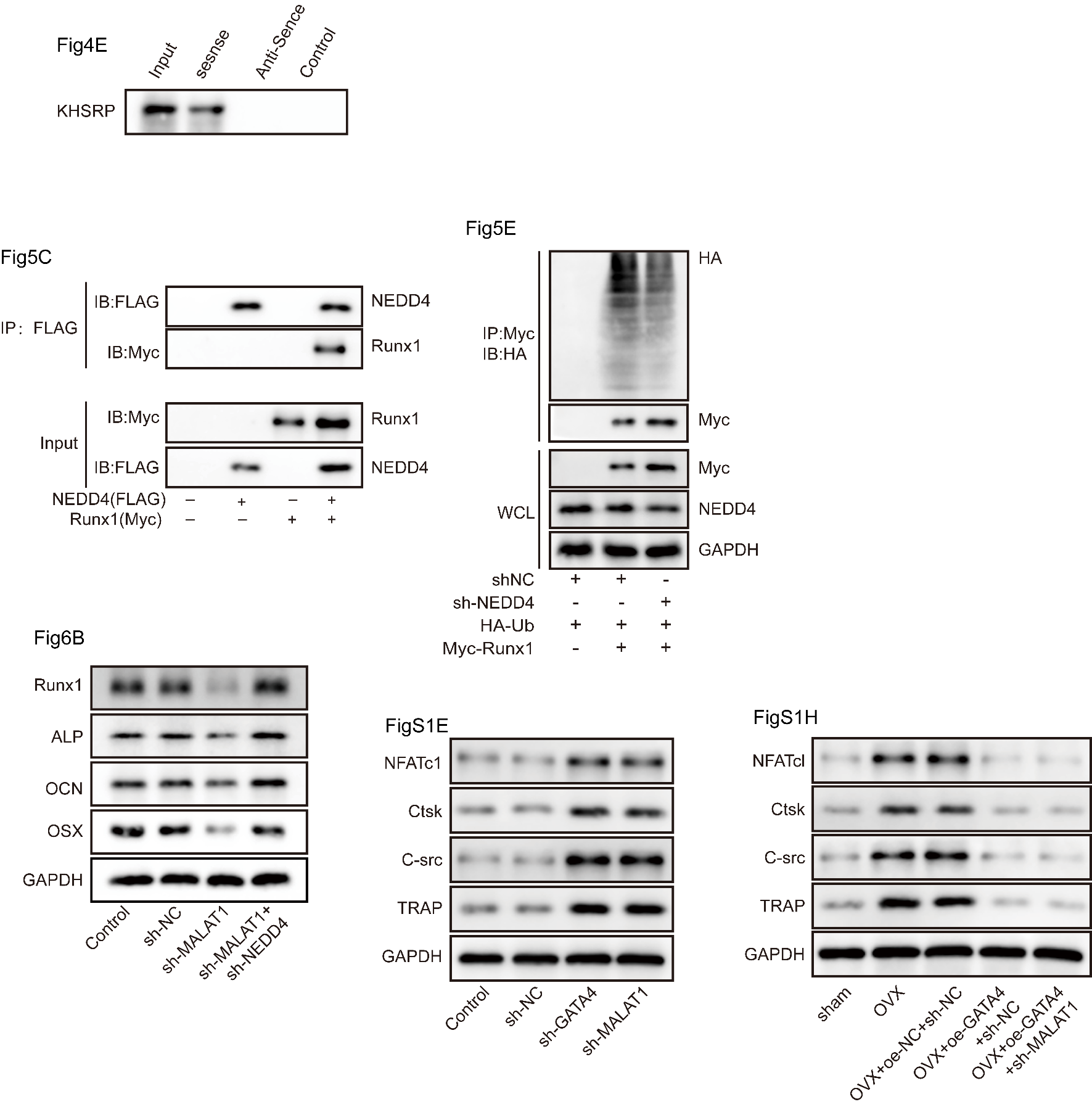


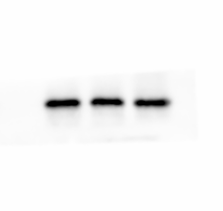


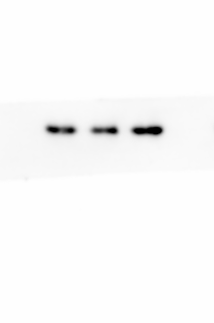

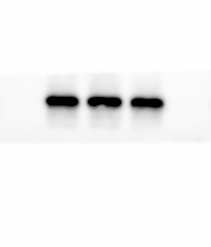

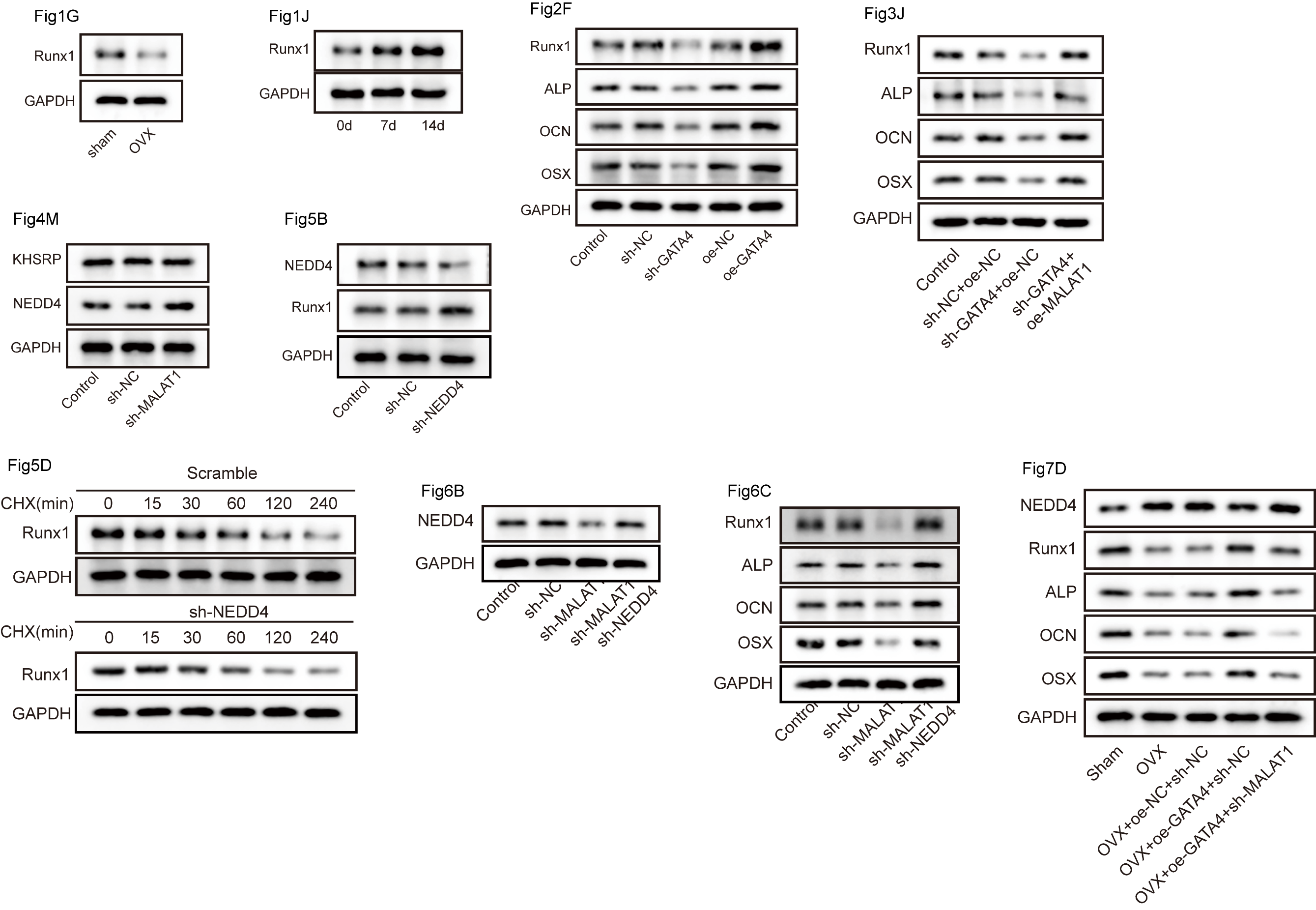


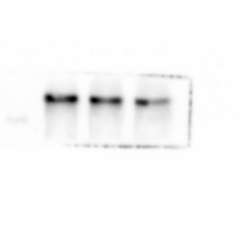


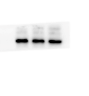

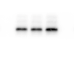

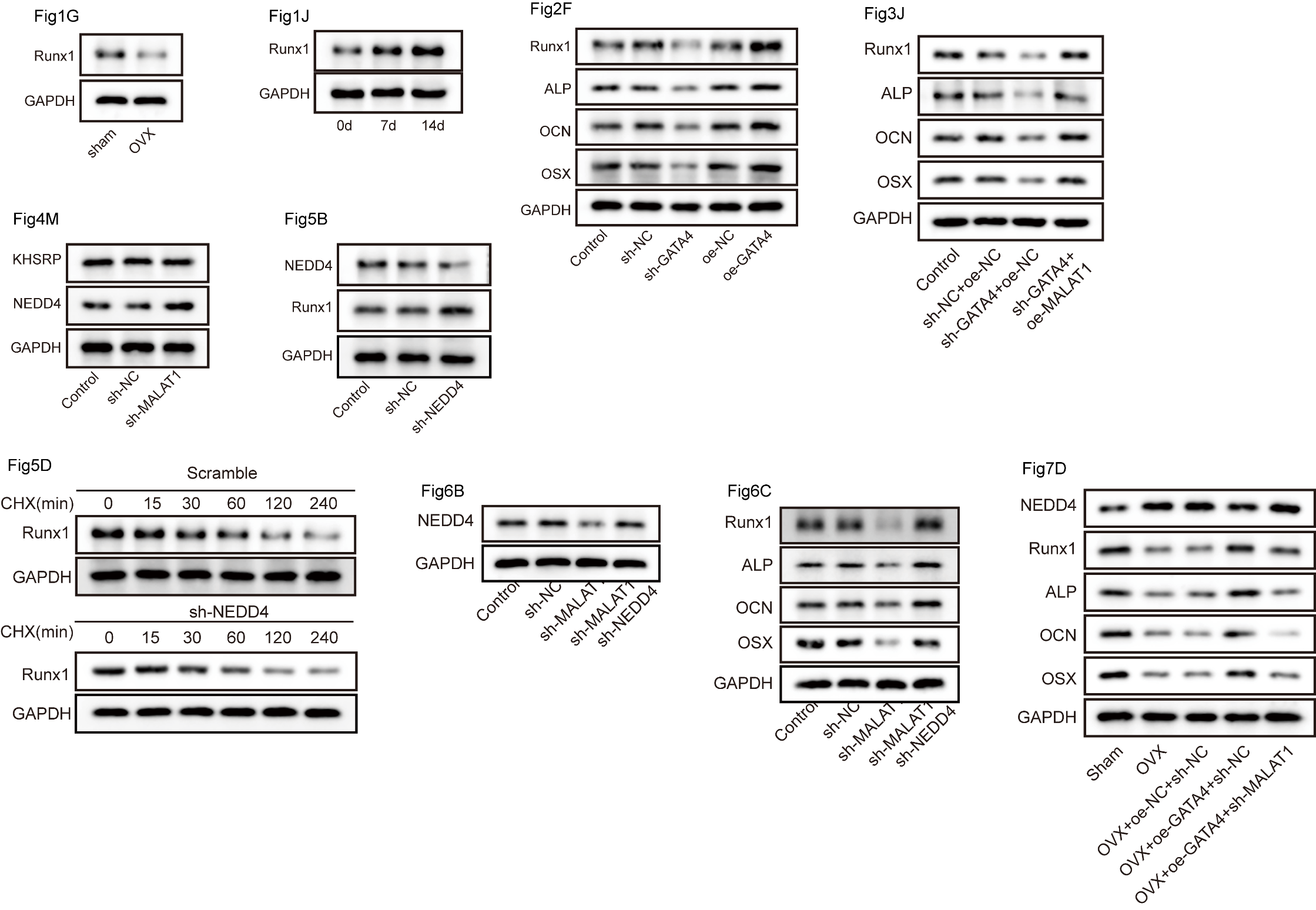


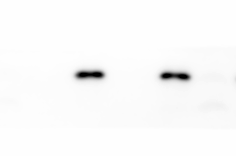


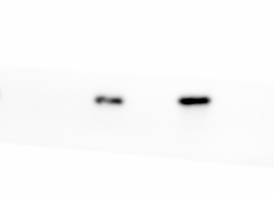

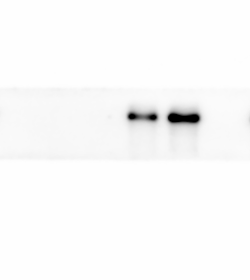

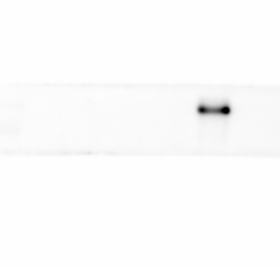

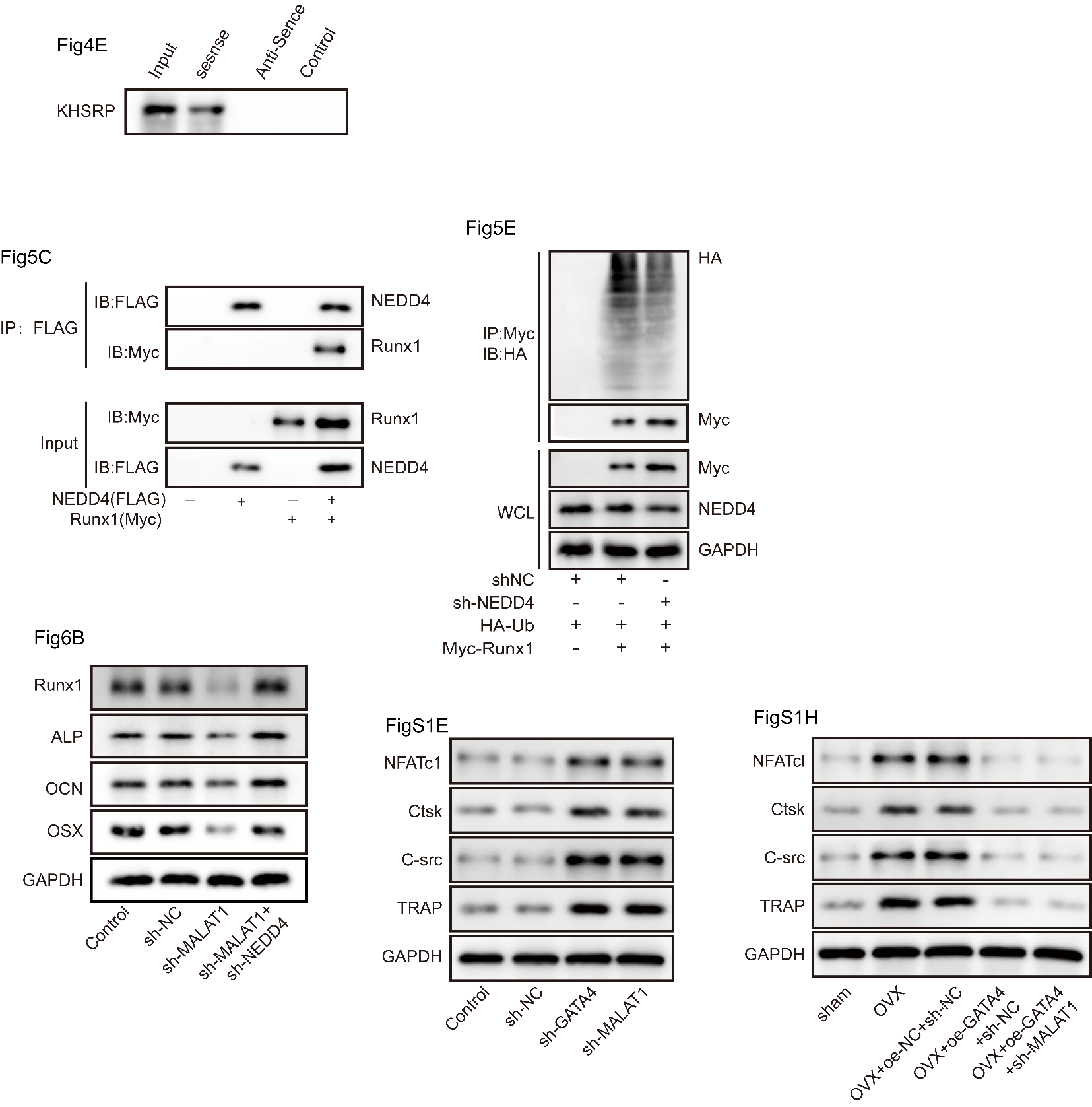


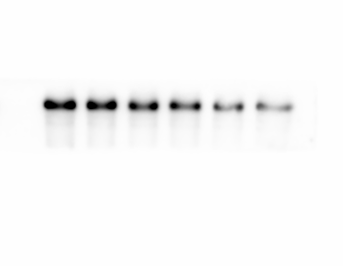


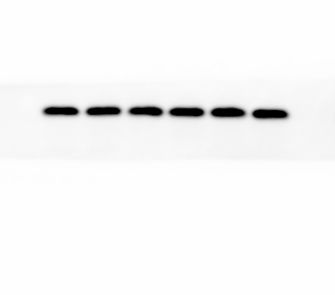

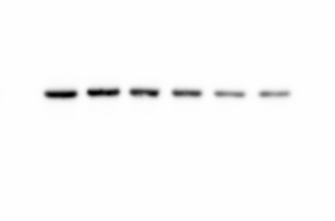

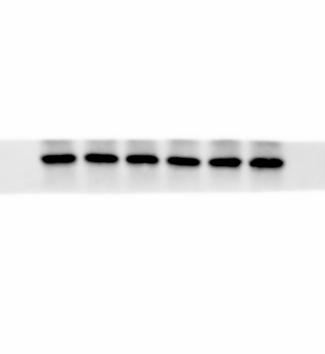

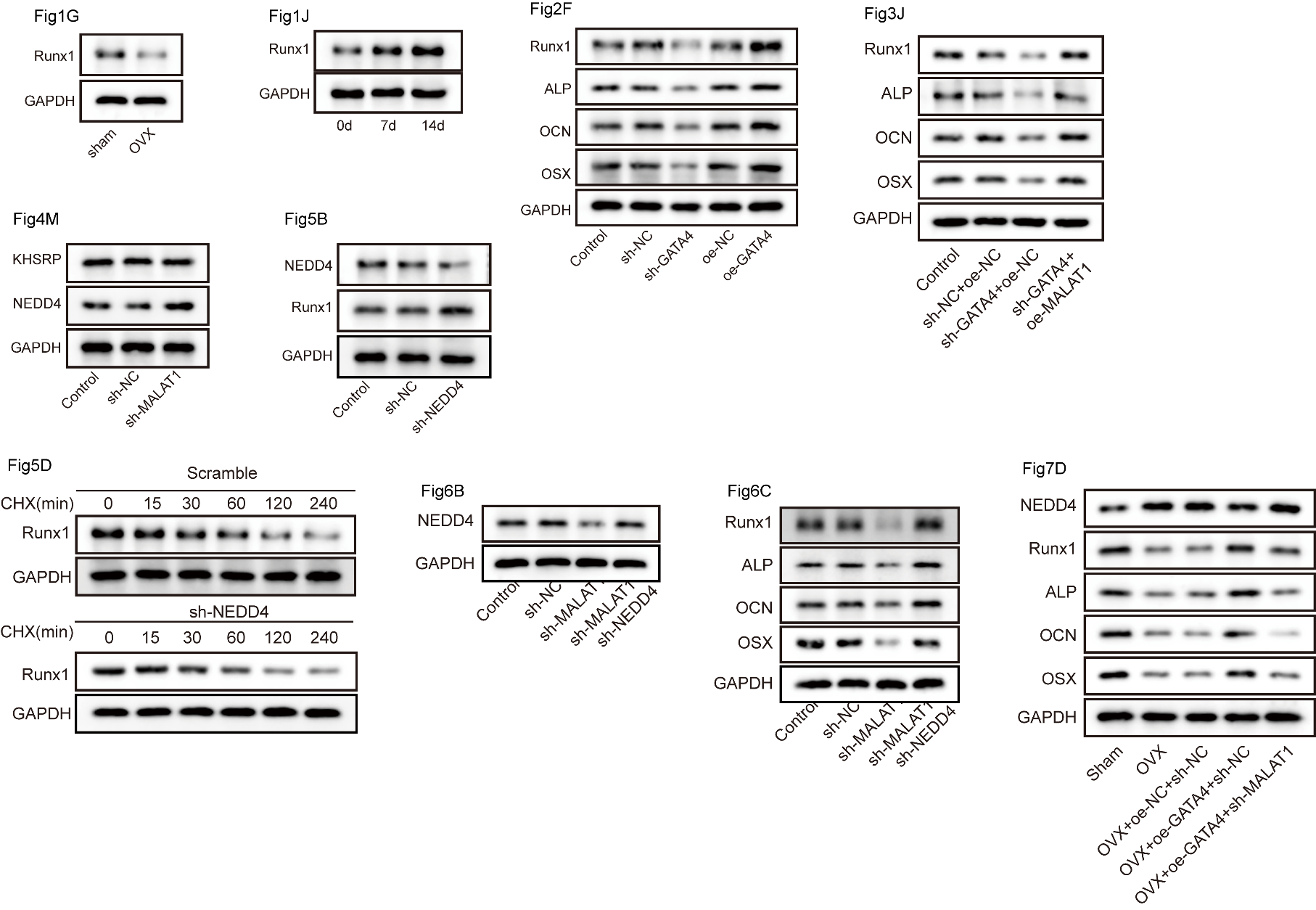


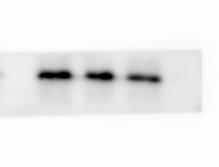

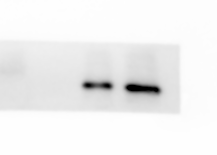

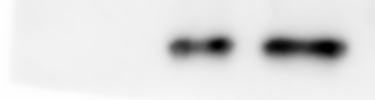

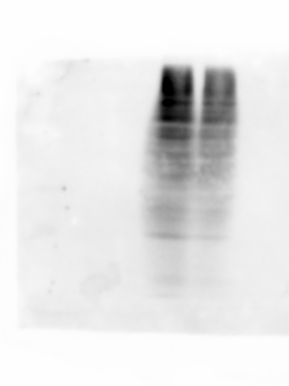

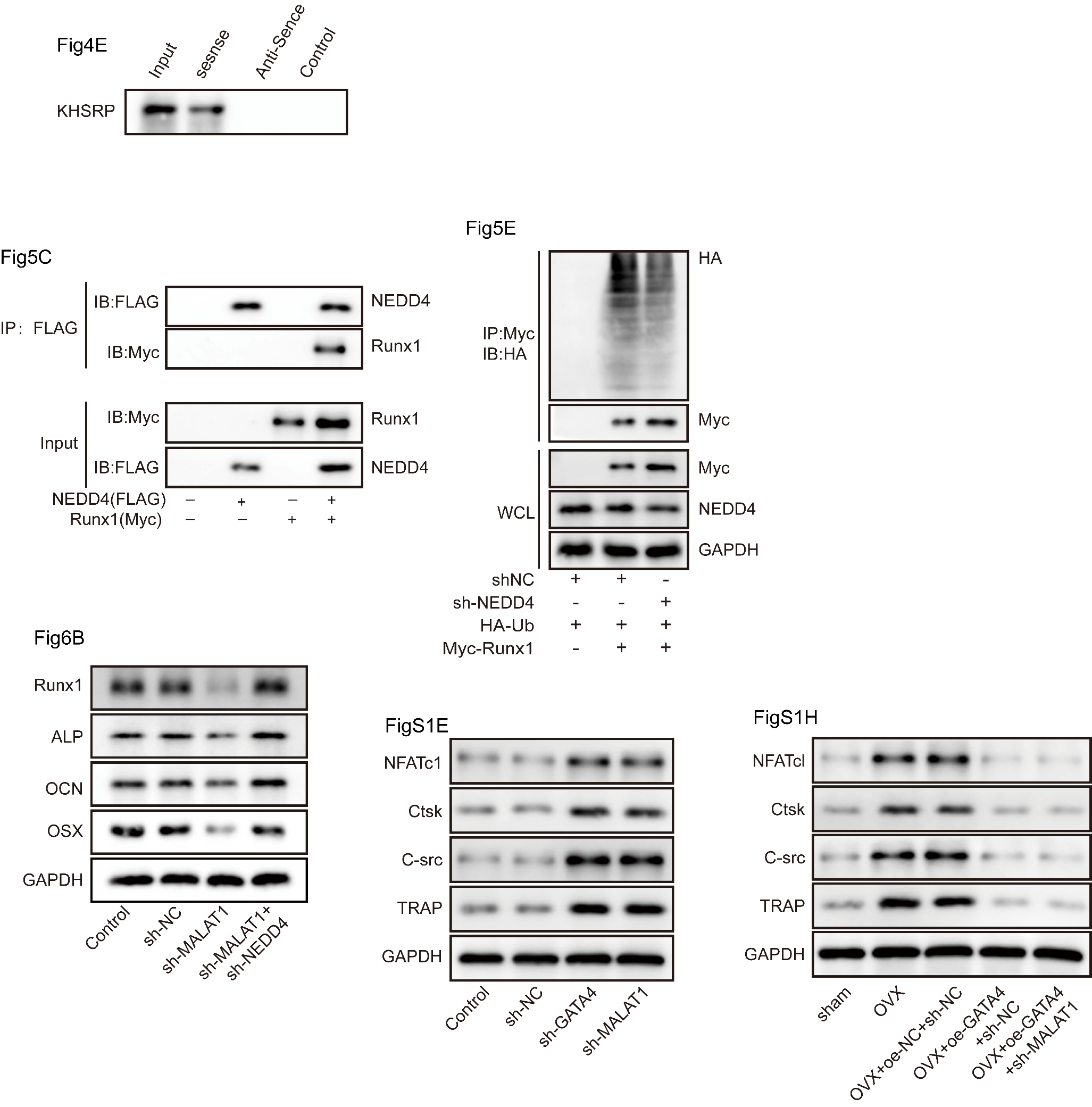


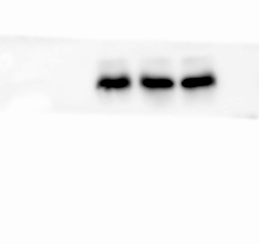


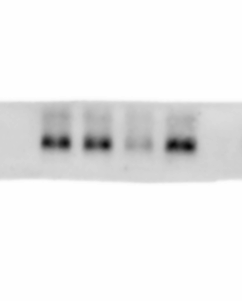


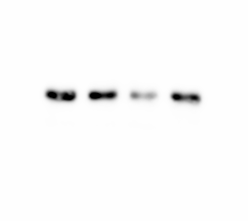

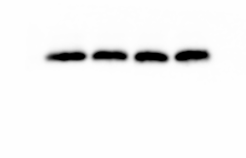

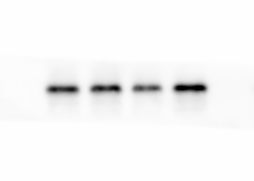

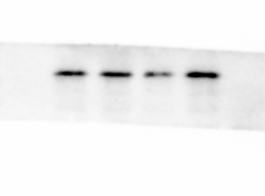

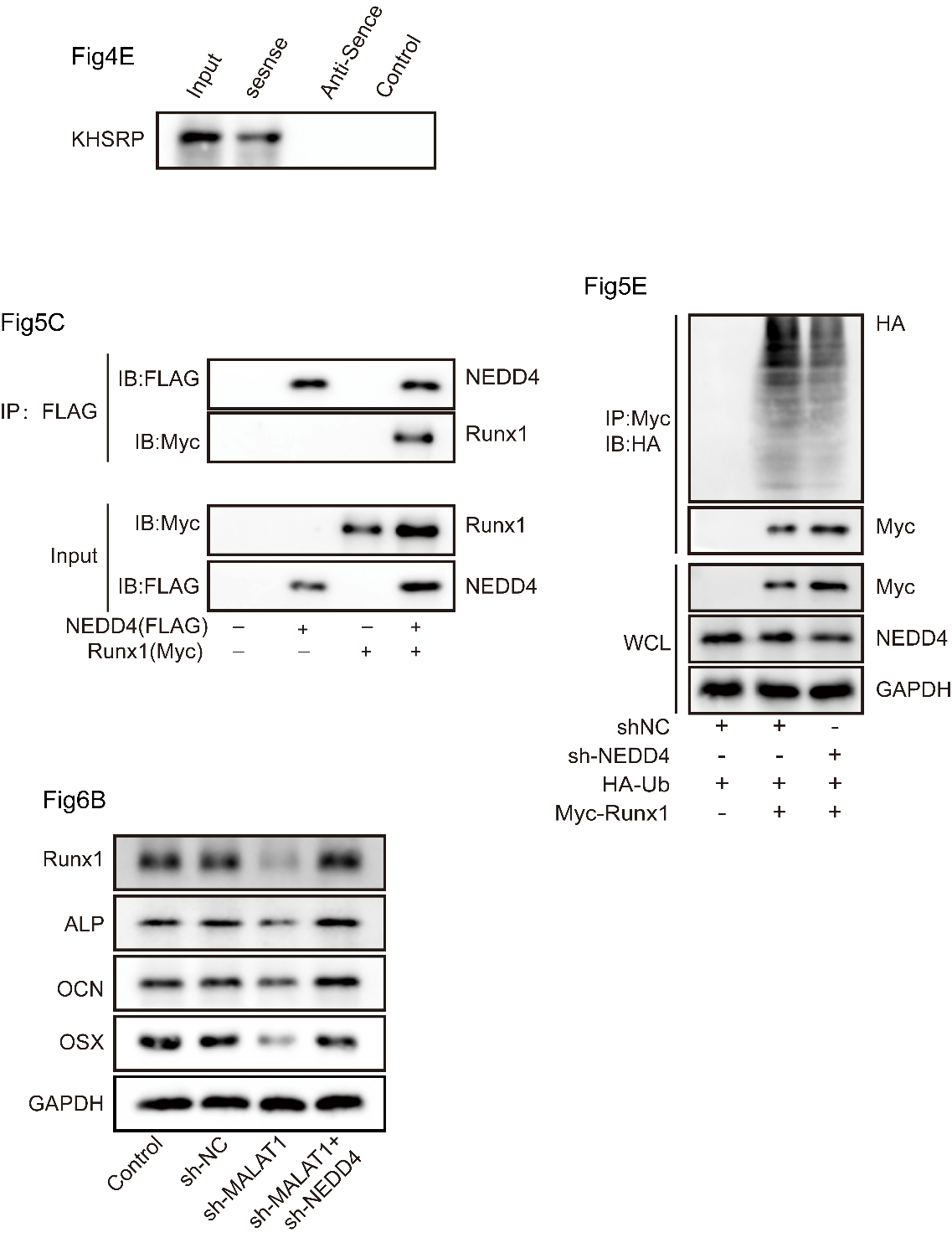


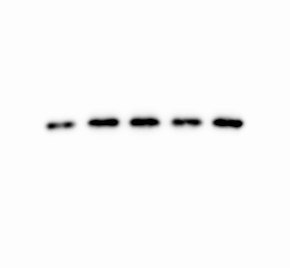


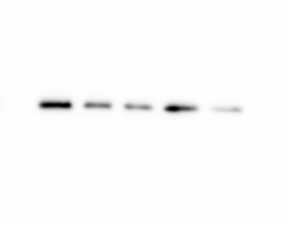

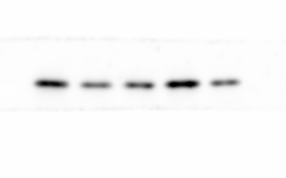

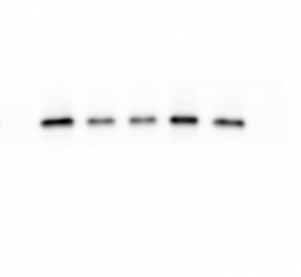

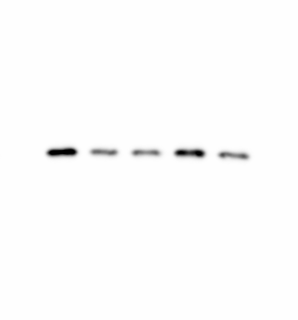

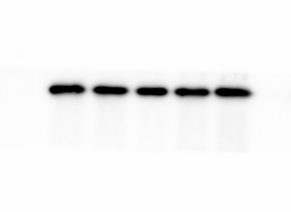

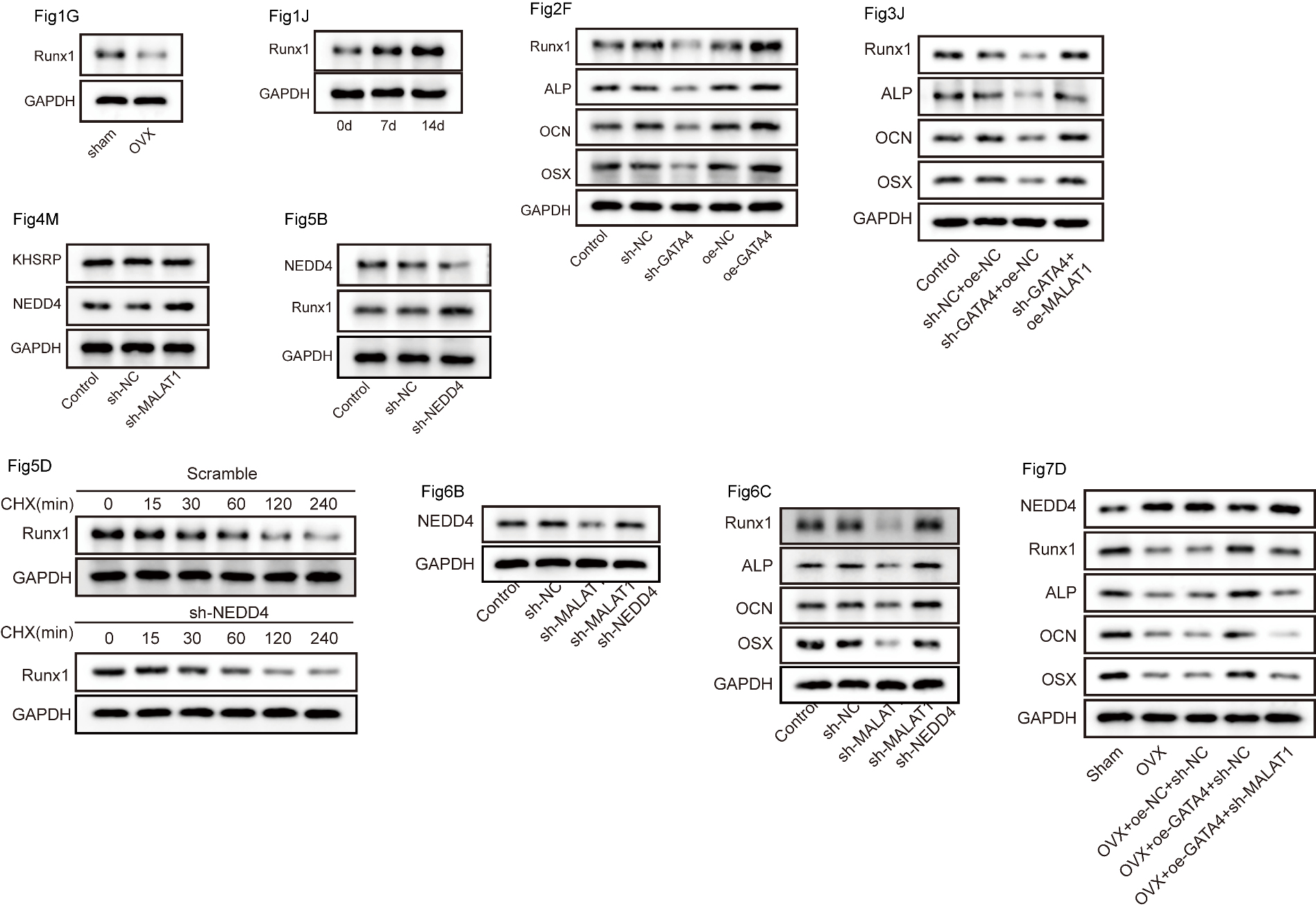


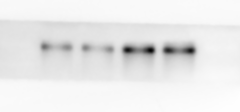


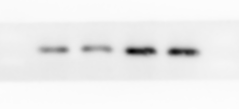


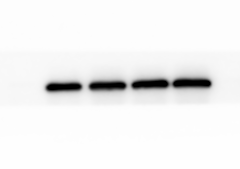

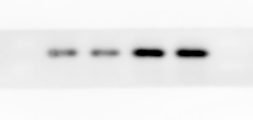

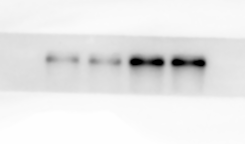

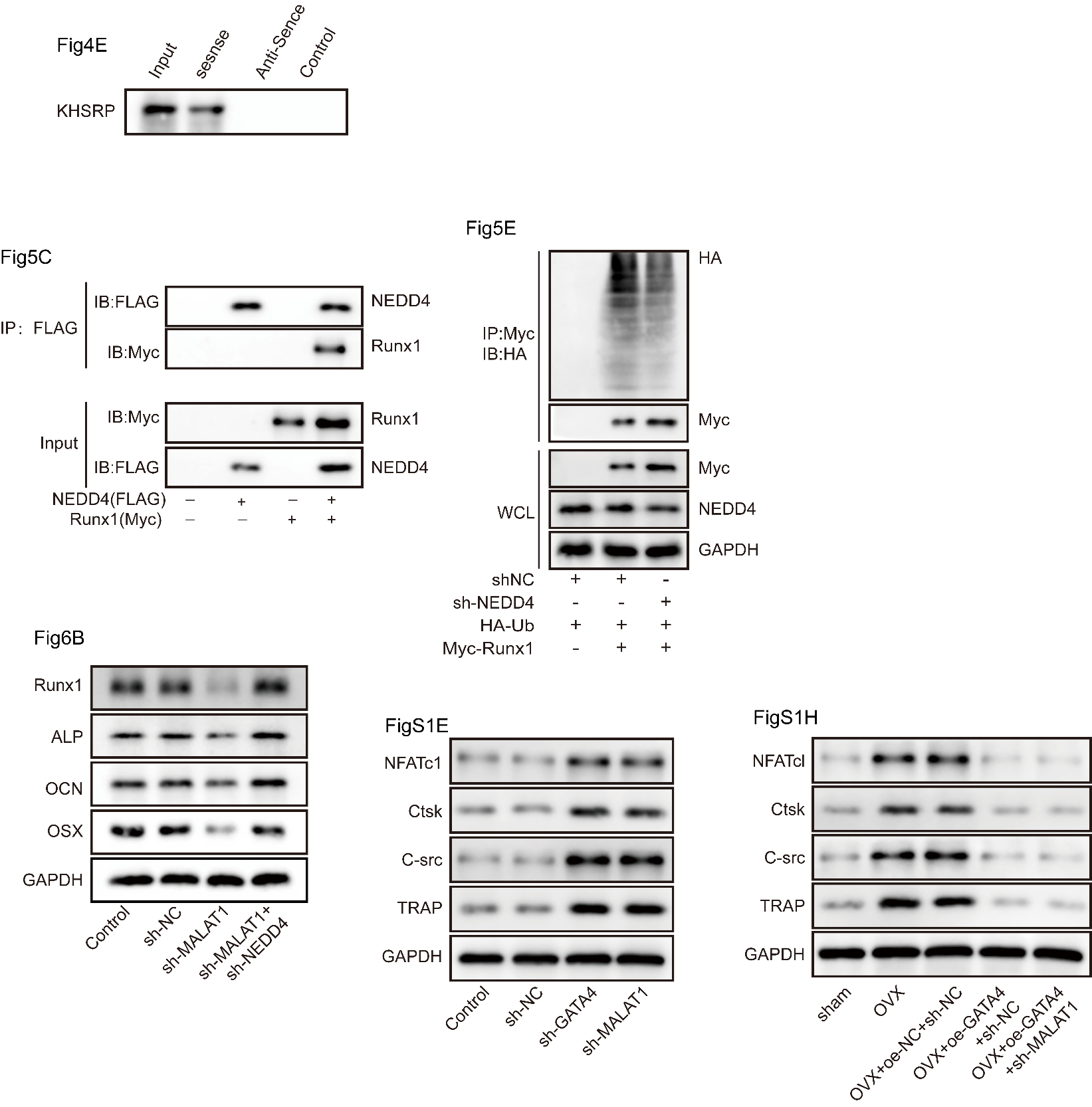

Supplement: Supplementary file 3 — Original Data File [file 41420_2023_1422_MOESM3_ESM.docx]
